# Dissolution of Phosphate and Precipitation of Carbonate in the Biomineralization of the Bivalve Shell *Limnoperna fortunei*

**DOI:** 10.3390/ani16162488

**Published:** 2026-08-10

**Authors:** Antonio Valadão Cardoso, Rodrigo Novaes Ferreira

**Affiliations:** 1 School of Design, State University of Minas Gerais (UEMG), Belo Horizonte 30140-091, MG, Brazil; 2 Microscopy Center, Federal University of Minas Gerais (UFMG), Belo Horizonte 31270-901, MG, Brazil

**Keywords:** biomineralization, mantle, bivalve, calcium phosphate, calcium carbonate, *L. fortunei*

## Abstract

The mantle of bivalves plays a fundamental role in shell formation and maintenance through biomineralization. Experiments performed on mantle–shell preparations using different experimental techniques revealed the presence of phosphorus (P)-containing compounds in the shell as the first mineral phase formed at the growing edge of the periostracum in the freshwater bivalve *Limnoperna fortunei*. The low P concentration in the shells suggests that phosphate is restricted to the growth regions and occurs at concentrations too low to be detected by techniques such as X-ray diffraction (XRD). Nevertheless, a crystal morphology closely resembling that of hydroxyapatite (HAp) was identified at the shell growth front, and a Ca/P ratio of 1.67, consistent with HAp, was determined in two of these regions. Fourier transform infrared (FTIR) spectroscopy also revealed the principal and most intense absorption band of the phosphate group (PO_4_^3−^) at 1024 cm^−1^. Enzymes such as carbonic anhydrase (CA), or proteins with equivalent functions, are likely involved in phosphate dissolution and the subsequent precipitation of calcium carbonate. In addition, the occurrence of calcium phosphate was confirmed in the shells of the marine bivalve *Perna perna*. Phosphate dissolution and carbonate precipitation during biomineralization suggest two important evolutionary advantages: first, the release and availability of phosphate, an essential nutrient for energy production and other metabolic functions; and second, the formation of a calcium carbonate shell, a structure indispensable for the protection, mechanical support, and survival of mollusks.

## Simple Summary

Shell growth in the freshwater bivalve *Limnoperna fortunei* was investigated through the study of its growth margins. Experiments performed on mantle–shell preparations using different experimental techniques revealed the presence of phosphorus (P)- and calcium (Ca)-containing compounds in the shell as the first mineral phase formed at the growing edge of the periostracum. Phosphate dissolution and carbonate precipitation at the biomineralization front suggest two important evolutionary advantages: first, the release and availability of phosphate, which has already been assimilated and is essential for energy production and other metabolic functions; and second, the formation of a calcium carbonate shell, a structure indispensable for the protection, mechanical support, and survival of bivalves.

## 1. Introduction

The control of golden mussel (*Limnoperna fortunei*, Dunker 1857) populations, which affect various economic activities in Brazil and in various parts of the world, has been the objective of different studies and research in Brazil [1]. One example is the growth and biomineralization of the shell, which, to be understood, requires investigation of the living body that produces it, especially the mollusk mantle.

The shells of bivalves are composites produced through a complex biochemical system that constructs microscopic structures in very short time, microscopic structures with mechanical properties that have deserved continuous attention [2,3,4,5]. These shells have properties that, for centuries, have been admired for their beauty and mechanics. However, only with the advent of 20th-century analytical instruments could their submicroscopic structures be partially understood [6]. Several possibilities for advancing the development of new composites inspired by biomineralization have been presented in recent years, aiming to achieve superior mechanical properties associated with sustainability in the production process [7,8].

Bivalve biocomposites are currently produced in a matrix of calcium carbonate (CaCO_3_), predominantly in its thermodynamic phases of aragonite and calcite. Thirty years ago, Lowenstam and Weiner (1989) [9] suggested that the demand for phosphorus (P) and its compounds in biological processes would be among the main reasons why bivalves and other invertebrates biomineralize shells and other rigid parts in CaCO_3_ composites instead of calcium phosphate.

Phosphorus and its compounds are essential in the biochemical processes of living organisms. On Earth, all life is supported by P compounds, even viruses, as their membrane and genetic heritage are phosphorylated. DNA and RNA nucleic acids are phosphorylated; the main sources of energy reserves in organisms, including adenosine triphosphate (ATP), are also phosphorylated. Many intermediate metabolites are phosphorylated compounds, and inorganic and organic phosphorus compounds are very suitable for biochemical syntheses and degradations. Several hypotheses have been raised about the biochemical option for P, including the ability of P compounds to be easily ionized at physiological pH [10].

Cook and Sheargold [11] suggested the occurrence of a massive phosphogenic event in the early Cambrian era which, associated with multiple environmental factors, resulted in a unique and remarkable phase in the evolution of life on the planet. It is considered that due to the availability of phosphates, shells during this period would mostly be made of calcium phosphate. A limiting factor for the growth of CaCO_3_ shells in phosphate-enriched oceans is the inhibitory effect that phosphate has on the nucleation and growth of amorphous or crystalline calcium carbonate phases by reacting with the substrate and blocking the carbonate growth [12]. The presence of ionic phosphate would be inhibitory for carbonate growth at the shell tip.

As a limiting nutrient for primary productivity, phosphorus is rapidly recycled in the environment, limiting its accumulation in sediments [13,14] despite the difference in sedimentation velocity as compared with calcium carbonate. Apatite, the most common phosphate mineral, has a molecular weight approximately 5 times higher than that of calcium carbonate. The higher molecular weight would favor sedimentation compared to calcium carbonate.

Thus, the advantage in producing phosphate shells was drastically reduced with the huge global expansion of the number of increasingly complex beings during the Cambrian period. Phosphorus is the eleventh most abundant element in the Earth’s crust, constituting approximately 0.1% of the Earth’s crust [15].

If the hypothesis of a more distant past consisting only of phosphate shells is correct, the inarticulate brachiopods can be considered “living fossils” because they are the only ones that currently build apatite-based shells containing also carbonate [16]. Such composite shells are dramatically different from those based on CaCO_3_. Starting with the concentrations of calcium phosphate (approximately 50–60%), which are much lower compared to the concentrations of calcium carbonate commonly found in bivalve shells (approximately 95%). Recent work [17] with brachiopod shells indicates the existence of hierarchical structures, at the nano, micro, and macro levels, besides very sophisticated mechanical and rheological behavior (viscoelastic). These features are especially attributed to the concentration of non-biomineralized organic components. The presence of an amorphous organic phase in the shells seems to be essential for its flexibility and its ability to adapt to the narrow and irregular holes in which these beings live. The complex hierarchical organization of chitin + protein [17,18] organic fibrils and lamellae in the shells of inarticulate brachiopods may thus indicate evolutionary competencies.

By studying the compositional profile of brachiopod shells, Watabe and Pan in 1984 [19] observed that the concentration of calcium phosphates was higher near the periostracum. The subsequent layers consist of organic fibrils (chitin + proteins) with biomineralized veins of calcium phosphate particles. The innermost part would be entirely organic. They attribute the lower levels of phosphate in the innermost parts to the slow metabolism of these animals. To understand the source of shell phosphate, the same authors then studied the pathways of Ca and P absorption in the brachiopods Glottidia pyramidata [20] using radioactive isotopes. They concluded that the phosphorus in the shells originates from the food digested by the animal. But the work also presents an intriguing fact: a much higher concentration of ^32^P was found in the mantle rather than in the shell.

There are many reports on the presence of phosphate compounds in bivalve shells, but always in concentrations of ppm or ppb. Bevelander and Benzer (1948) [21] were among the pioneers in investigating the calcification process in the bivalves *Atrina rigida* (*Pinna*) and *Pedalion alatum*. They looked inside the mantle and were seeking to understand the internal dynamics until the materials reached its outer surface. In intentionally damaged shells, the authors observed that the first biomineralization granules were of calcium phosphate. Scholars, for over a century (W. Biedermann, 1914, cited by Bevelander and Benzer 1948), have already wondered why the mantle of modern bivalves contained calcium phosphate while the shell is built with calcium carbonate. Through the results obtained from the use of radioactive phosphorus (^32^P), Belevander in 1952 [22] suggested that the phosphorus present in the mantle is subepithelial and related to phosphorylation processes, whose function the author found to be unrelated to shell growth.

The presence of an extrapallial space proposed by A. de Waele in 1930 [22] between the mantle and the inner face of the shell made the discussion about the phosphate present in the mantle even more suitable since the biomineralization activity would occur in that space and, therefore, no longer directly from the pallial surface of the mantle [23]. In the investigation of the components present in the extrapallial fluid, phosphate was not included [24]. The attention devoted to the so-called extrapallial space as the site of chemical reactions resulting in the precipitation of CaCO_3_ seems to conflict with the nanometric dimensions [25,26] that this space would have, for example, in bivalves. In these animals, as far as we know, there is no experimental evidence to confirm whether this extrapallial space has a constant thickness or even if it is continuous. Moreover, the rheology of fluids, such as water and aqueous suspensions, confined between two hydrophilic surfaces of nanometric thickness, has specific physical characteristics [27]. Thus, the so-called extrapallial fluid, which would fill this space, must have very different properties from a Newtonian fluid. Anyway, the scarce number of experimental measurements of the fluid and its cavity seems to raise more questions than answers [28].

Studies on mantle–shell interfaces in freshwater bivalves are notably scarce [28,29,30]. Nonetheless, some authors suggest, based on personal observations [29], that in freshwater mussels, the extrapallial space appears to be larger and more pronounced, entirely filled with extrapallial fluid. However, no conclusive evidence has been presented to support this claim.

*Limnoperna fortunei* (golden mussel) is a freshwater bivalve and an exotic species in South America, where it was introduced during the last decade of the twentieth century [31,32]. With very high reproduction rates [33,34], the adult golden mussel has millimeter-thick shells that, when dry, fracture and crumble under finger pressure. Observed under an electron microscope, the golden mussel shell presents a complex and hierarchical structure [35] with a first layer of calcite adhered to the periostracum, followed by a nacreous layer of aragonite tablets, and the innermost part possessing a prismatic layer also of aragonite.

In the present work, we focus on the analysis of shell edges and their interaction with parts of the mantle. A long and thorough investigation using scanning electron microscopy SEM coupled with energy-dispersive spectroscopy (EDS) technique on the complex structure of the mantle and its surface in contact with the *L. fortunei* shell was carried out to investigate the occurrence of calcium phosphate in shell growth regions. Other experimental techniques, namely ICP-OES, WDXRF, XRD, FTIR, and FTIR-ATR were employed to confirm the presence of phosphorus (P) and phosphates at the growing shell edge. To verify if the occurrence of phosphorus and phosphate would be unique to *L. fortunei*, shell samples of *P. perna* were tested using the same techniques.

## 2. Materials and Methods

### 2.1. Specimen Collection and Preparation

Approximately 300 specimens of *Limnoperna fortunei* (golden mussel) were collected from the reservoir of the Volta Grande Hydroelectric Power Plant (21°46′ S, 42°32′ W), Minas Gerais, Brazil, in January (summer), when mean daily temperatures ranged from 21 to 30 °C. In the laboratory, the animals were maintained in aquaria under controlled conditions of pH, electrical conductivity, water temperature, ammonia, nitrite, nitrate, phosphate, calcium, alkalinity, and other water-quality parameters, at an ambient temperature of 24 °C. Routine monitoring of body weight, shell size, and general health was performed throughout the maintenance period. Legal authorization for the collection, maintenance, and use of the animals for scientific research was granted by the Brazilian Ministry of the Environment (MMA), through the Chico Mendes Institute for Biodiversity Conservation (ICMBio), under a permit issued by the Biodiversity Authorization and Information System (SISBIO).

Fifty specimens of the marine mussel *Perna perna* (Linnaeus, 1758) were purchased from a local market. The mussels originated from aquaculture farms in the region of Santa Catarina Island (27°22′–27°50′ S, 48°25′–48°35′ W), Santa Catarina, Brazil. In the laboratory, the *P. perna* specimens were kept frozen until use in subsequent experiments.

For both species, the shells were separated from the soft tissues and initially washed under running tap water in a laboratory sink until all organic and inorganic residues (e.g., clay, sand, and other mineral particles) had been completely removed. The shells were then air-dried and stored in desiccators until further use. Sample preparation was carried out at the Laboratory of the Center for Bioengineering of Invasive Species in Hydroelectric Power Plants (CBEIH), located at Biominas, Belo Horizonte, Brazil. Preparation of the mantle–shell complexes for scanning electron microscopy (SEM) and energy-dispersive X-ray spectroscopy (EDS) analyses was performed at the Center of Microscopy of the Federal University of Minas Gerais, also located in Belo Horizonte, Minas Gerais, Brazil.

### 2.2. Quantitative Chemical Composition by ICP-OES

For the quantification of selected chemical elements present in shell samples of *L. fortunei* (golden mussel) and *Perna perna*, the inductively coupled plasma optical emission spectrometry (ICP-OES) technique was used. This quantification was performed on three different occasions, in the year 2016, using an ICP-OES instrument, Perkin Elmer-Optima 3000 (Perkin Elmer, Shelton, CT, USA). In 2022 and 2023, the metals present in shell samples were quantified again using a PerkinElmer Optima DV 8300 ICP-OES instrument (Perkin Elmer, Shelton, CT, USA). The methodology used was validated according to the procedures recommended by official bodies (ISO-7 cleanroom and ISO-5 laminar flow hood). The tests were carried out at CIT-Senai in Belo Horizonte, MG. The detection limit of both instruments was 0.2 μg/L [36].

### 2.3. Chemical Composition by WDXRF

For the quantification of selected chemical elements present in samples of *L. fortunei* (golden mussel) and *Perna perna* shells, the wavelength dispersive X-ray fluorescence spectrometry (WDXRF) technique was used, Malvern Panalytical, model Axios Max (Malvern Panalytical, Malvern, UK). The tests were performed at CIT-Senai in Belo Horizonte, MG.

### 2.4. X-Ray Diffraction (XRD)

For the X-ray diffraction analysis of the crystalline phases present in the edges of the shells of *L. fortunei* and *P. perna*, a multipurpose diffractometer, Malvern Empyrean Panalytical model (Malvern Panalytical, Malvern, UK) equipped with a Cu X-ray tube and a two-dimensional PIXEL 2 × 2 detector was used. The experimental parameters of the measurement were 45 kV voltage, 40 mA cathode current, and a 2θ range of 20–90°. The quantification of the phases was performed based on the Inorganic Crystal Structure Database (ICSD). To perform the tests, the *L. fortunei* shells were carefully washed with deionized water and a brush, then air-dried.

After the cleaning step, the edges of the *L. fortunei* shells were easily broken manually with the aid of stainless-steel tweezers. Irregular pieces of a few mm in size were obtained by this procedure. To collect the edges of the *P. perna* shells, it was necessary to use a steel clamp due to higher resistance to fracture of the shells.

All XRD analyses were performed on shell powder prepared by grinding the shells with a mortar and pestle.

### 2.5. Scanning Electron Microscopy (SEM) with Energy-Dispersive Spectroscopy (EDS)

Preparations and analyses of *L. fortunei* shells using scanning electron microscopy (SEM) were conducted at the Federal University of Minas Gerais Microscopy Center (CM-UFMG) using two instruments: (a) FEI Quanta 3D FEG dual-beam (FEI Quanta, Hillsboro, OR, USA) with backscattered electrons (BSE) detector and energy-dispersive X-rays (EDS); and (b) APREO-2C ThermoFisher (ThermoFisher, Waltham, MA, USA) with standard BSE and EDS detectors.

Ten specimens of *L fortunei* were selected, treated with buffered Karnovsky fixative solution, dehydrated with graded alcohol concentrations and dried using the critical-point drying Leica EM CPD030 (Leica, Wetzlar, Germany). Two types of preparations were investigated: a-Shells with all internal parts of the animal; b-Shells with the mantle attached. For the preparation of the mantle–shell assembly, the specimens were opened using of a stainless-steel spatula, and the soft tissues of the bivalve were carefully dissected to ensure that the mantle–shell assembly could be clearly analyzed. After this initial stage, the samples were coated with a 50 nm graphite layer and kept in a low humidity desiccant cabinet before and after the SEM sessions.

Fourteen sessions of 2 h each were carried out to investigate different parts of the *L. fortunei* shell and mantle. The maps of the chemical elements carbon (C), oxygen (O), calcium (Ca) and phosphorus (P) show slightly different colors in different figures due to SEM-EDS sessions carried out on different dates.

### 2.6. FTIR and FTIR-ATR Spectroscopy

FTIR and FTIR-ATR tests were performed on samples of the edges of *L. fortunei* and *P. perna* shells with identical protocols. To perform the tests, both *L. fortunei* and *P. perna* shells were carefully washed with deionized water and a brush, then air-dried. After this cleaning procedure, the edges of the *L. fortunei* shells were broken manually with the aid of stainless-steel forceps. Irregular pieces with millimeter sizes were obtained by this procedure. To collect the edges of the *P. perna* shells, it was necessary to use a steel clamp due to the higher resistance to fracture of the shells. Irregular pieces of these edges were pulverized for the FTIR tests. For the FTIR-ATR tests, entire irregular pieces of the edges of the shells were used.

Infrared absorption spectra were measured on a Perkin-Elmer FTIR model RX I spectrometer (Perkin Elmer, Shelton, CT, USA) between the wavenumber range 400–4500 cm^−1^. The powder samples were mixed with KBr and then pressed into a pellet. All measurements were carried out at room temperature. Powder spectra were recorded at a resolution of 2 cm^−1^, whereas ATR-FTIR spectra were recorded at a resolution of 1 cm^−1^.

### 2.7. Carbonic Anhydrase Assay

The presence of the enzyme carbonic anhydrase in *L. fortunei* shells was assessed using the protocols, with modifications, of Miyamoto [37] (hereafter referred to as Method 1) and Fuchs [38] (method 2), following the preparation and cleaning procedures proposed by Sakalauskaite [39]. The method involves changing the pH from 8.3 (method 1)/8.2 (method 2) to 7.3 (method 1)/6.5 (method 2) due to the activity of the metalloenzyme, which catalyzes the interconversion of CO_2_ and water into a bicarbonate ion and a proton. This method was developed by Freeman and Wilbur [40] and detailed by Wilbur and Anderson [41].

A total of 40 g of *L. fortunei* shells was ground and left for 24 h in a 2% sodium hypochlorite solution for cleaning. After air-drying, 20 g was immersed in a 0.5M EDTA pH 8.0 solution (PHT, Belo Horizonte, Brazil) and 20 g in 4% acetic acid (Vinagre Dicasa, Contagem, Brazil) for 6 days for demineralization, under continuous stirring. The supernatants were collected, diluted with distilled water, and stored at 2 °C for 24 h, then centrifuged at 12,000 rpm for 20 min and stored again under refrigeration.

Half of the supernatant demineralized with EDTA received 2 mM ZnCl_2_ to neutralize the metal chelating effect, followed by centrifugation and refrigerated storage. The other half was not.

Bovine carbonic anhydrase (CAB, Sigma-Aldrich, St. Louis, MO, USA) at 12 mg/L in distilled water cooled to 2 °C was tested as a reference. The volume used in each assay was 1 mL of solution.

Following protocol **method 1** with modifications, 10 mL of pH 7 phosphate buffer (Dinâmica, São Paulo, Brazil) and 5 mL of the demineralized suspension and 6 drops of phenol red (Dinâmica, São Paulo, Brazil) were added to a 100 mL glass beaker, which was placed in a Styrofoam container with cold water and ice. The addition of pH 10 buffer (Dinâmica, São Paulo, Brazil) raised the pH to 8.3. A digital pH meter (Akso, São Leopoldo, Brazil) with temperature control was immersed in the beaker, and then, to initiate the assay, 5 mL of ice-cold water (0–2 °C), previously bubbled with pure CO_2_ for 30 min using a flowmeter (Voha Instr., Changzhou, China), was added.

Using **method 2** [38] with modifications, 40 mL of 0.1 M Tris buffer solution (Orion, São Paulo, Brazil), adjusted to pH 8.2 with the addition of H_2_SO_4_, was placed in a 100 mL glass beaker with a digital pH meter (AKSO, São Leopoldo, RS, Brazil) and a stainless-steel CO_2_ diffuser (commercialized supplied BoB, São José dos Campos, SP, Brazil). The test solutions were then added (5 mL of the demineralized suspensions or 1 mL of CAB), without exceeding 40 mL of liquid. The test was carried out at room temperature (23–25 °C, Belo Horizonte, Brazil) and started with the injection of CO_2_ into the beaker using a flowmeter (Voha Instr., Changzhou, China) set at 400 mL/min of CO_2_.

The results of the carbonic anhydrase assays are presented in the Supplementary Material.

## 3. Results

### 3.1. SEM-EDS of the Shell Edges

**Figure 1:**
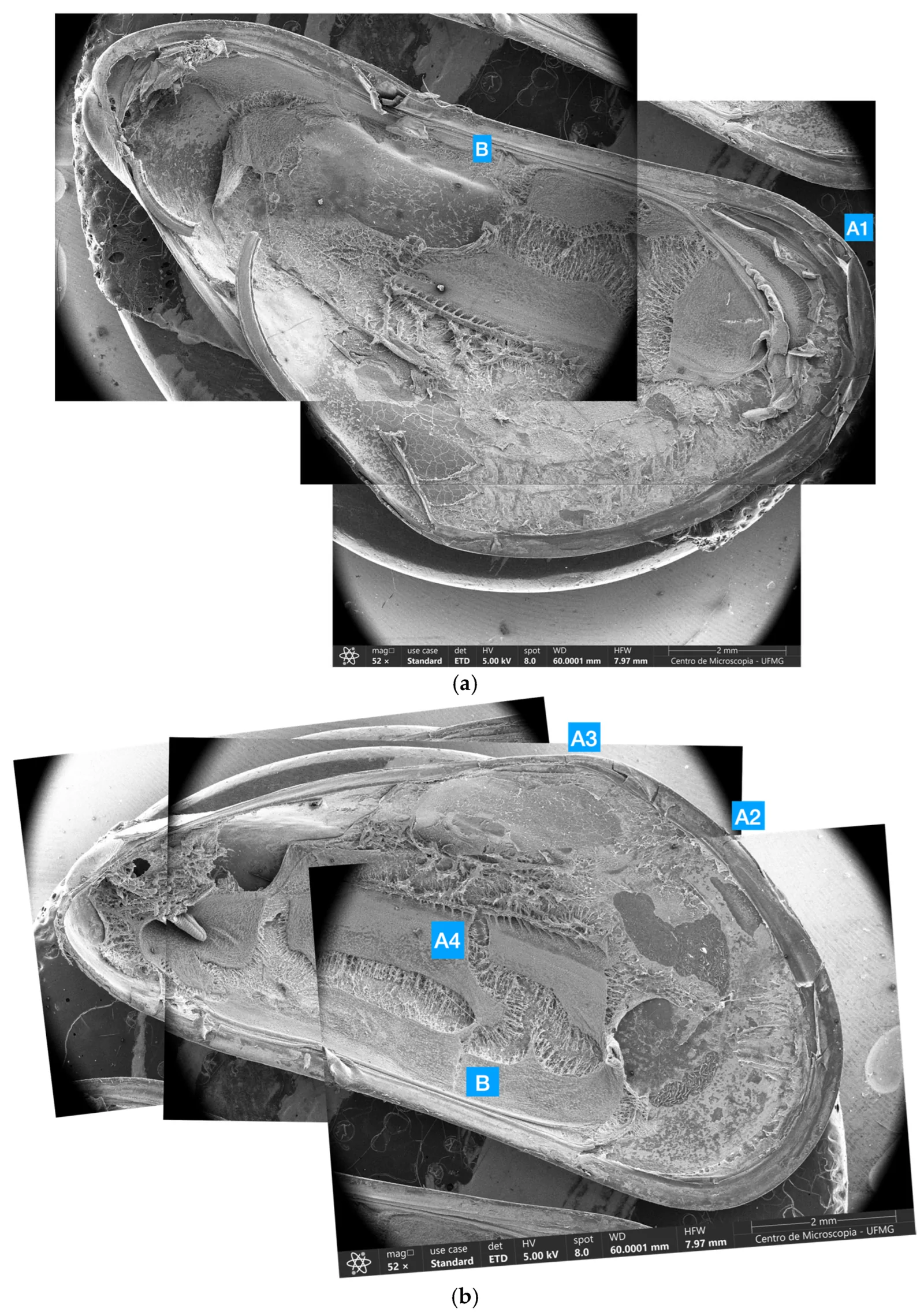
(**a**,**b**) Two *L. fortunei* shells with portions of the mantle attached. The image montage highlights regions A1–A4 described in the text. The periostracum covers the shell edge, and regions A1, A2, and A3 correspond to fractures in this layer that exposed the underlying biomineralized layer for investigation. In (**a**), the hinge joining the two valves is partially detached from the shell edge on the lower side of the mount. Region A4 corresponds to tears in the mantle. Immediately above A4, the mantle edges are visible on both shells. Of the three mantle folds (inner, middle, and outer), only the inner fold (B) is clearly visible. The periostracum is secreted by the outer fold, which appears as a ledge adjacent and parallel to the inner fold. The inner surface of the mantle is intact in some regions and torn in others, exposing the mantle interior, the inner side of the outer mantle surface in contact with the shell, and, in some areas, the biomineralized shell surface itself. The intact mantle–shell assembly was prepared by opening the specimens with a stainless-steel spatula and carefully dissecting the soft tissues while preserving the mantle attached to the shell (see Figure S2 in the Supplementary Material).

The SEM images in Figure 1 were composed to show the entire area of the two shells with portions of the mantle of the bivalve *L. fortunei*. SEM images of the two shells with the viscera of the golden mussel are presented in Supplementary Material Figure S2. From the images in Figure 1, it is possible to locate and visualize the interior of the mantle (see also Supplementary Material Figure S3), as evidenced by tears in the mantle. The inner part of the mantle has a three-dimensional structure of interconnected fibers or fiber aggregates, forming a micrometer-scale sponge-like structure, which has already been reported in previous work on the mantle of other bivalves [42]. The shells are approximately 2–3 cm long. Region A1 is marked at the shell edge in Figure 1a, whereas regions A2, A3, and A4 are marked in Figure 1b. These areas were studied in detail to obtain images, acquire elemental maps, and perform semiquantitative analyses using EDS. Region A4 is one of the locations where tears in the mantle make it possible to investigate its interior. Just above A4, structures known as mantle edges can be observed on both shells [43]. Of the three folds at the mantle edge (inner, middle, and outer), we identified the inner fold, which is visible, outlined, and marked with the letter B in Figure 1a,b. The periostracum is secreted by the outer fold, the beginning of which can be seen in the SEM image as a ledge adjacent and parallel to the inner fold. The inner surface of the mantle is intact in some areas and torn in others. In the torn areas, it is possible to observe the interior, including the inner side of the mantle’s outer surface, which is in contact with the shell. In some areas, the biomineralized surface of the shell itself is visible.

Below we describe each of these regions of the shell edge and the results we found.

### 3.2. Shell Edge A1

**Figure 2:**
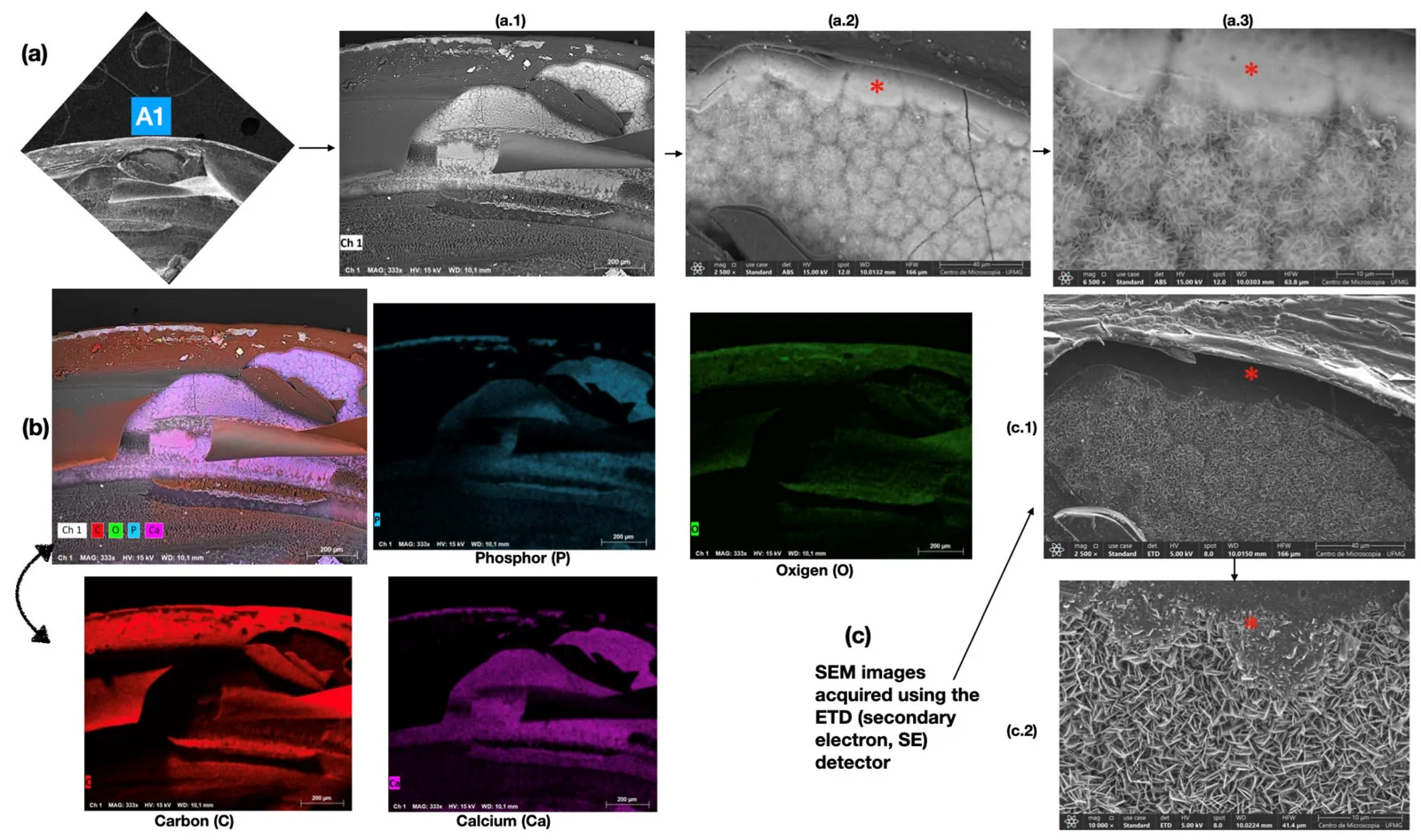
(**a**) SEM images of region **A1**. Images (**a.1**–**a.3**), acquired sequentially at increasing magnifications using the ABS detector, focus on the aggregates located immediately beneath the periostracum of *L. fortunei*. (**b**) the image on the right shows energy-dispersive spectroscopy (EDS) elemental maps highlighting the main chemical composition of the region: carbon (C) throughout the periostracum, calcium (Ca) and phosphorus (P) in the aggregates, and oxygen (O) in both the periostracum and the aggregates. (**c**) Images (**c.1**,**c.2**), acquired using the ETD detector, provide another view of the aggregates, which consist of a calcium phosphate compound (P, O, and Ca). Image (**c.2**) clearly shows two-dimensional growth in the form of plates. Images (**a.2**) and (**c.1**) were acquired at the same magnification as (**a.3**) and (**c.2**), respectively. The red asterisks (**a.2**,**a.3**,**c.1**,**c.2**) indicate apparent coalescence of the upper surfaces of these morphologies. A similar feature is observed in Figure 3d and is highlighted by a red asterisk.

The **A1** region is located at the inner tip of the *L. fortunei* shell (shown in detail in Figure 2). The shell tips are the regions that record the most recent growth of the bivalve. The images in Figure 2 were acquired using two types of SEM detectors: BSE/ABS (backscattered electron/annular backscatter) to observe the surface and its topography (Figure 2a), and ETD (Everhart–Thornley detector) to obtain images (Figure 2c) generated by secondary electrons originating from deeper regions of the beam–sample interaction.

**Figure 3:**
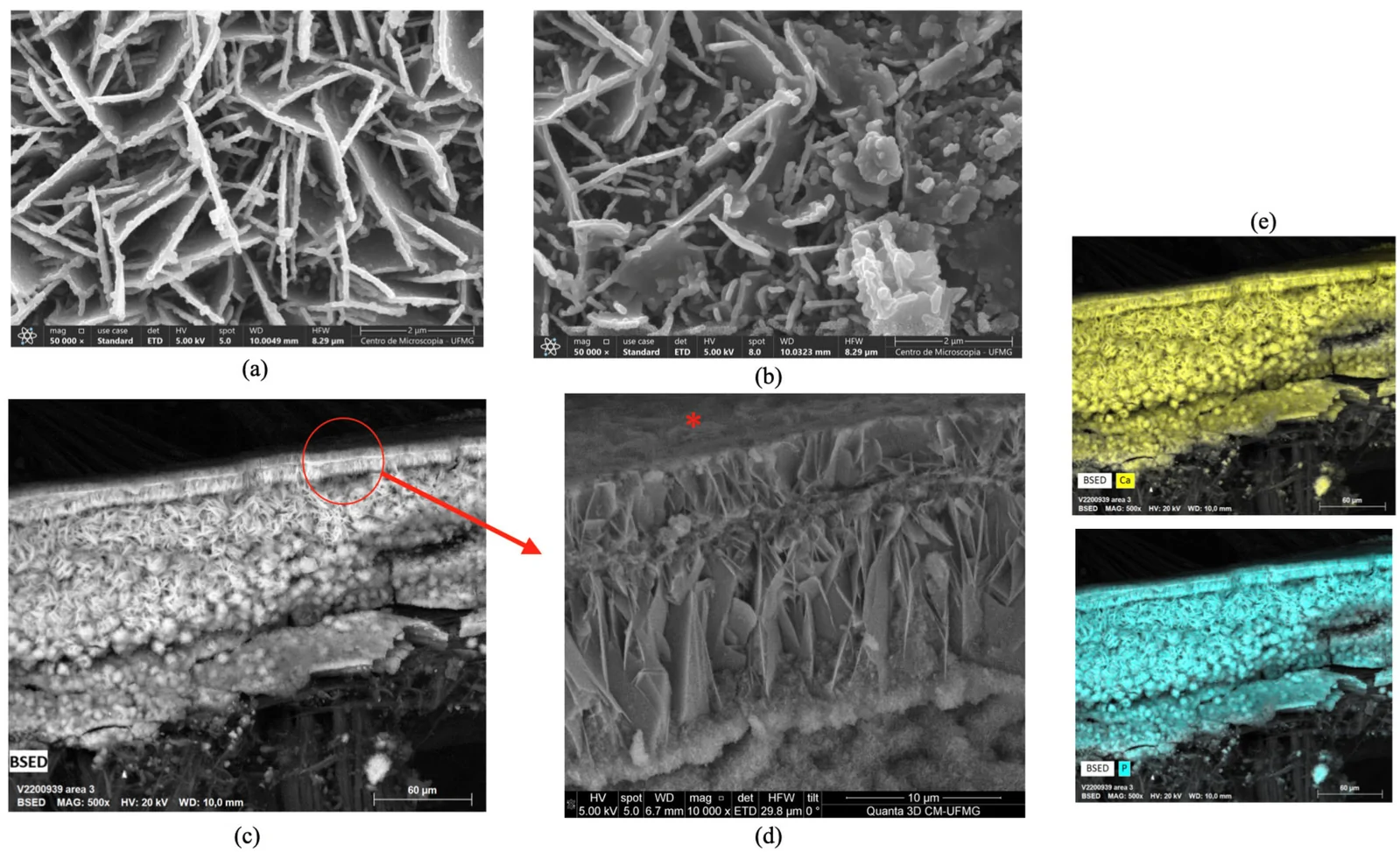
(**a**,**b**). Detailed view of the aggregates at the shell growth front (Figure 2c). Nanometer-sized spherical particles are observed in both images. Panel (**c**) shows the mineralized layer previously attached to the periostracum, and the detail in panel (**d**) shows, in lateral view, the same plates observed in frontal view in panel (**a**). Panel (**e**) shows the calcium (Ca) and phosphorus (P) elemental maps corresponding to the same region as the aggregates shown in panel (**c**). The red asterisk in panel (**d**) indicates that the surface above the plates appears to be coalesced. A similar feature can be observed in Figure 2(a.2,a.3,c.1,c.2), where this coalesced surface also appears above the plates. Images in panels (**a**,**b**) are from one *L. fortunei* specimen, whereas panels (**c**–**e**) are from another *L. fortunei* specimen.

The periostracum appears on the inner surface at the shell tip, where it has been secreted by the mantle (visible at the bottom of Figure 2(a.1)). The image of region **A1**, at the inner tip of the shell, is shown at the beginning of the image sequence in Figure 2a, which focuses on the aggregates immediately beneath the periostracum of *L. fortunei* at increasing magnifications (Figure 2(a.1–a.3)). In Figure 2(a.1), these aggregates can be observed at the outermost shell tip, indicating that biomineralization extends to the shell margin. Figure 2(a.2,a.3) show the morphology of these aggregates more clearly. Figure 2b presents elemental maps of the main chemical elements present in this region. Energy-dispersive spectroscopy (EDS) provides semiquantitative chemical information from the characteristic X-rays emitted by the chemical elements present within the region irradiated by the electron beam. Carbon (C) clearly marks the periostracum, an organic film secreted by the mantle fold (at the bottom of Figure 2b, shrinkage during sample drying caused it to detach from the mantle edge). Oxygen is present in both the periostracum and the biomineralized aggregates. The main elements detected in the aggregates are phosphorus (P), calcium (Ca), and oxygen (O), indicating that they consist of a calcium phosphate compound (P, O, and Ca). The aggregates are well individualized in the higher-magnification image shown in Figure 2(a.3). The ETD images in Figure 2c show the structure of the individual aggregates in greater detail. Figure 2(c.2) shows plate-like structures growing perpendicular to the image plane. These plates appear to grow through the addition of nanometer-sized spheroidal particles (Figure 3).

In both Figure 3a,b, two-dimensional growth of plates composed of a phosphate compound can be observed. These plates appear to grow through the adhesion of phosphate nanoparticles. The interaction between adjacent plates seems to indicate that the surface of one plate promotes the nucleation and growth of another. The nanometer-sized spheres are possibly amorphous and may crystallize as they organize into plate-like structures.

### 3.3. Shell Edge A2

**Figure 4:**
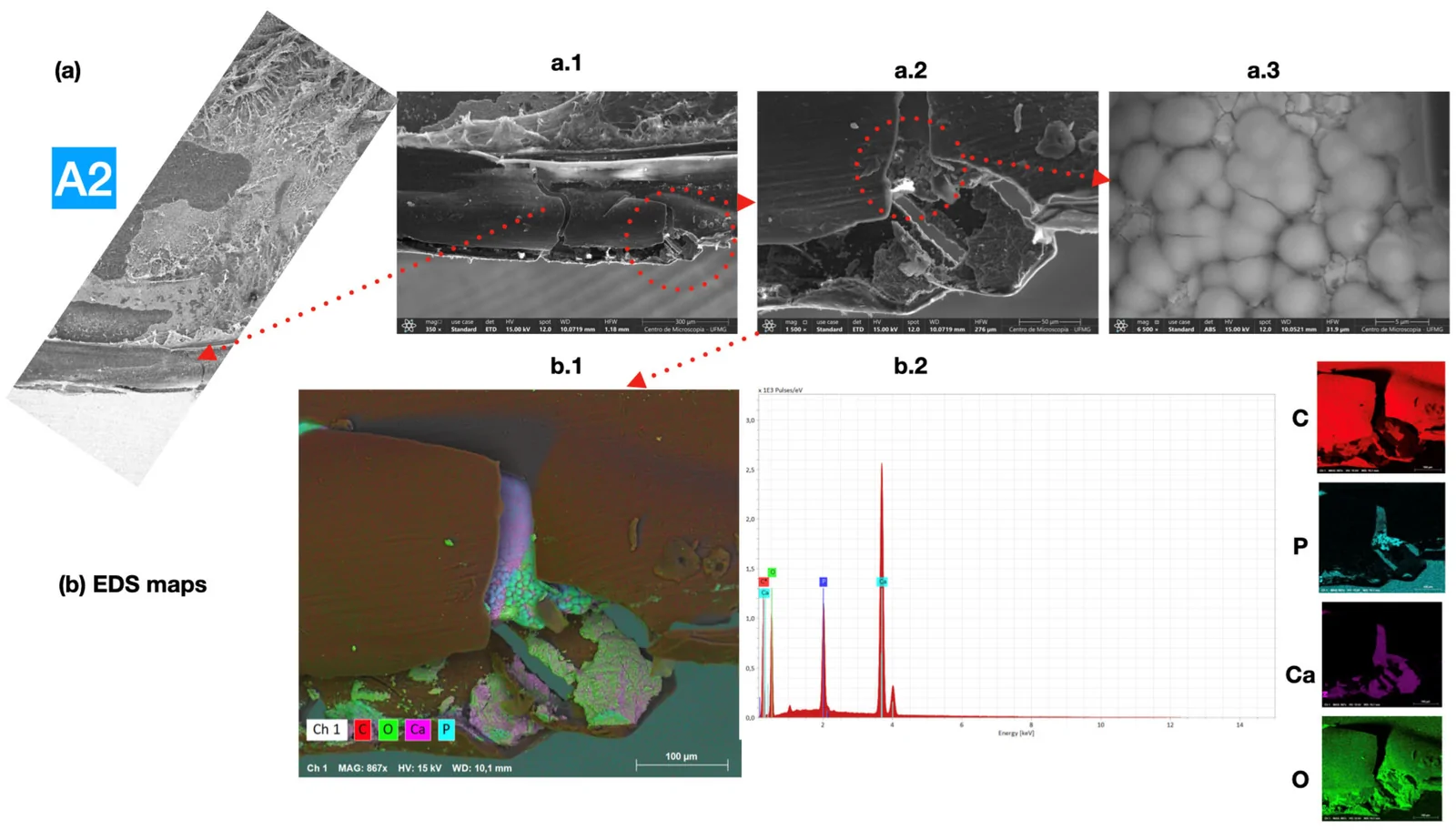
(**a**) SEM images of region **A2**, shown in the sequence of images (**a.1**–**a.3**), focusing on the aggregates immediately beneath the periostracum of *L. fortunei*. (**b**) Image (**b.1**) shows elemental maps highlighting the main chemical composition of the region, displayed on the right side of the figure: carbon (C) throughout the periostracum region, calcium (Ca) and phosphorus (P) in the aggregates, and oxygen (O) in both the periostracum and the aggregates. The concentrations of the main elements are shown in Table 1, and the corresponding EDS spectrum is presented in image (**b.2**).

**Area A2**, located in the shell shown in Figure 1b, is presented in detail in Figure 4. The sequence of images a.1, a.2, and a.3 focuses on the region containing aggregates immediately beneath the periostracum of *L. fortunei*. Aggregates whose composition, as estimated by EDS (Figure 4(b.1)), indicated the presence of phosphorus (P), oxygen (O), and calcium (Ca) are observed immediately beneath the periostracum. The spectrum showing the concentrations of these elements is presented in Figure 4(b.2). Elemental maps showing the distribution of these chemical elements are presented on the right side of the figure. Carbon is detected throughout the periostracum, calcium and phosphorus mark the aggregates, and oxygen is detected in both the periostracum and the aggregates. The semiquantitative concentrations of these elements are given in Table 1.

**Figure 5:**
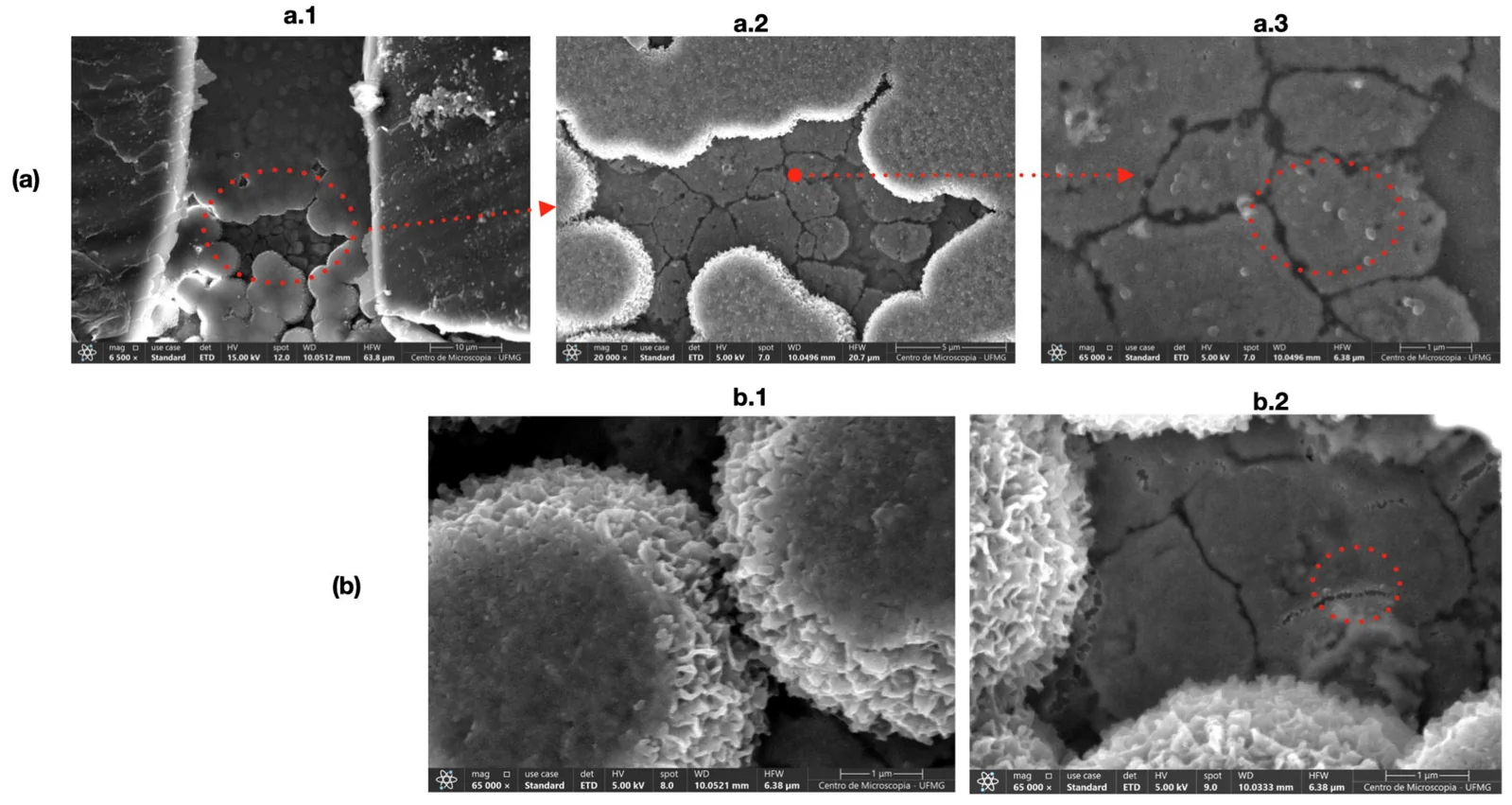
(**a**,**b**). Detailed view of the morphology of the aggregates in region A2, as shown in the previous figure. Immediately beneath the periostracum, aggregates identified by EDS as containing phosphorus (P), oxygen (O), and calcium (Ca) are present (Figure 4(b.1)). These aggregates (shown in detail in (**b.1**)) tend to form a continuous layer through coalescence ((**a.1**,**a.2**)). Immediately beneath these aggregates, the nacreous layer appears, as shown in (**a.3**). Nanometer-sized particles are visible in (**a.3**,**b.2**) and are circled in red. The origin, direction of movement (downward, sideways, etc.), and chemical composition of these nanoparticles are unknown.

The images in Figure 5 show the morphology of the aggregates presented in the previous figure. Immediately beneath the periostracum are aggregates identified by EDS (Figure 4(b.1)) as containing phosphorus (P), oxygen (O), and calcium (Ca). These aggregates (shown in detail in Figure 5(b.1)) tend to form a continuous layer by coalescence (Figure 5(a.1,a.2)). The structural organization of these aggregates, whether crystalline or amorphous, could not be characterized. Immediately beneath these aggregates appears what seems to be the nacreous layer of the *L. fortunei* shell, shown in Figure 5(a.3). This nacreous layer is strongly marked by calcium (Ca) in Figure 4(b.1) but appears not to contain phosphorus (P). Finally, nanometer-sized particles are observed in Figure 5(a.2,b.3) and are circled in red. The origin (whether detached from or attached to the aggregates), direction of movement (downward, sideways, etc.), and chemical composition of these nanoparticles remain unknown.

### 3.4. Shell Edge A3

**Figure 6:**
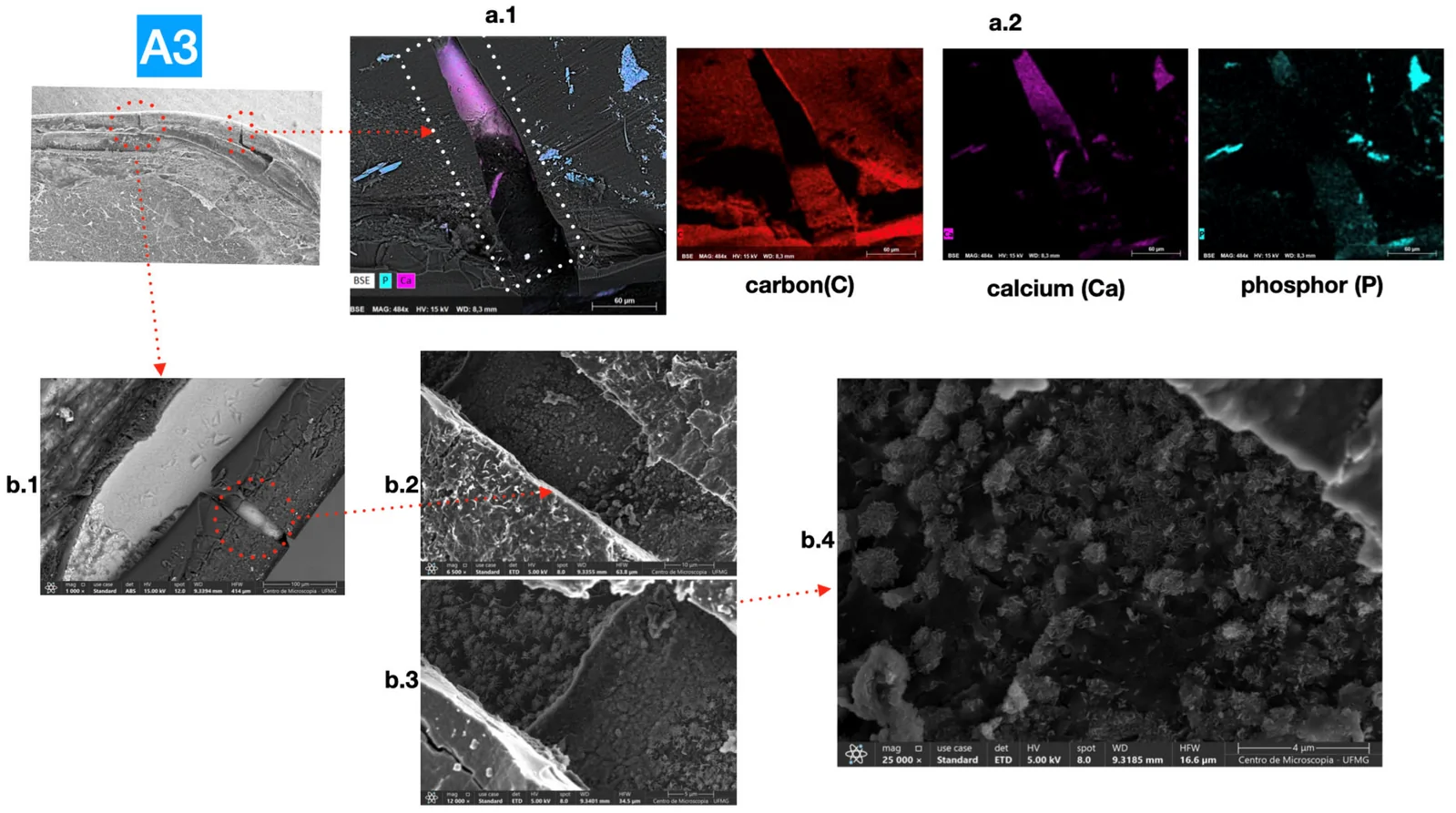
SEM images of region **A3**, where two fractures are present at the shell edge (circled in red). (**a.1**) shows an energy-dispersive spectroscopy (EDS) elemental map highlighting the main chemical composition of the region, whereas (**a.2**) presents elemental maps of carbon, calcium, and phosphorus, showing the distribution of these elements throughout the periostracum region and the aggregates. The semiquantitative concentrations of these elements are presented in Table 1. The sequence of images in (**b.1**–**b.4**) focuses on the fracture at the left shell edge. The aggregates containing P, O, and Ca have the same morphology as those in region **A1** (see Figure 2 and Figure 3). (**b.4**) shows that these aggregates tend to coalesce (see the red asterisks in Figure 2(a.2) and Figure 3d).

**Region A3** has two fractures at the shell edge (circled in red in the A3 image shown in Figure 6). Figure 6(a.1) shows energy-dispersive spectroscopy (EDS) elemental maps highlighting the main chemical composition of the region, with the elemental maps in Figure 6(a.2) showing carbon throughout the periostracum, and calcium and phosphorus marking the aggregates. The semiquantitative concentrations of these elements are presented in Table 1.

Figure 6(b.1–b.4) focus on the fracture at the left edge. The aggregates containing P, O, and Ca have the same morphology as those in region A1 shown in Figure 3. Figure 6(b.4) shows that these aggregates tend to coalesce and form continuous plates, as also observed in Figure 3b.

### 3.5. Mantle–Nacre and Region A4

**Figure 7:**
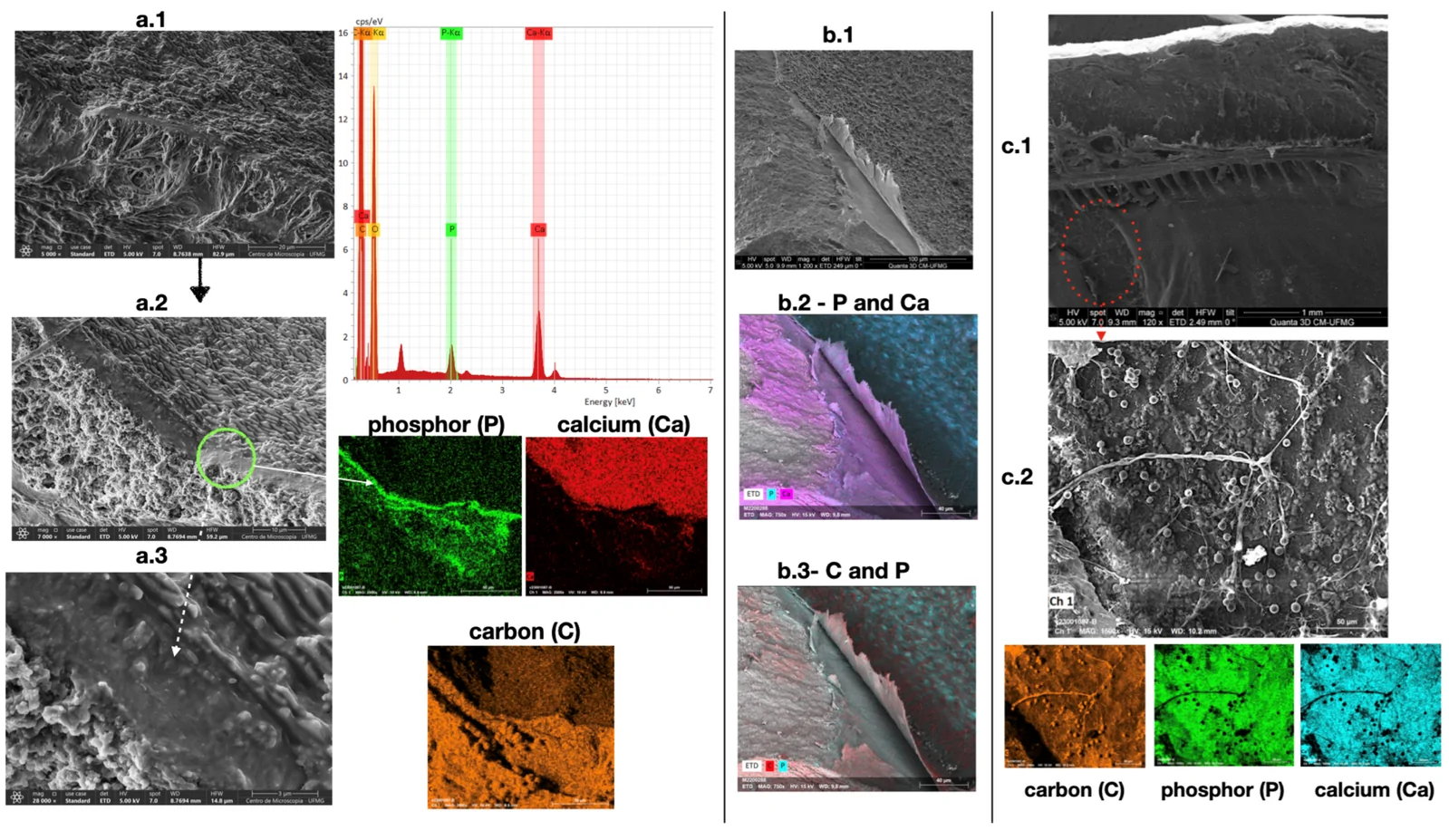
SEM images of regions where the mantle layer overlaps the nacreous layer of the *L. fortunei* shell (**a.1**–**a.3**). The elemental maps show a continuous band enriched in phosphorus (P) and calcium (Ca). Carbon (C) clearly outlines the mantle, including spherical structures that are possibly mucus. Images (**b.1**–**b.3**) illustrate the presence of P, Ca, and C at the mantle–nacre interface. Image c.1 shows an area of the shell containing the mantle with part of the bivalve musculature (region A4 in Figure 1b). Torn regions of the mantle (outlined in red in (**c.1**)) are shown in detail in (**c.2**). Image (**c.2**) shows the mantle layer in contact with the shell. The carbon map outlines several structures, including internal mantle fibers and possibly mucus spheres. In addition, phosphorus (P) and calcium (Ca) are present throughout the inner surface of the mantle.

In addition to the shell edges, we investigated areas where the mantle was located directly above the nacre, as shown in the SEM images in Figure 7(a.1–a.3). The elemental maps show a continuous band composed of phosphorus (P) and calcium (Ca). The thickness of the phosphate layer, measured by SEM, is approximately 3 μm (see Supplementary Material Figure S6). The calcium phosphate layer is composed of nanoparticles of varying sizes, as shown in the higher-magnification image in Figure 7(a.3). Beneath this layer lies the nacreous layer, which is composed of calcium carbonate. In Figure 5, calcium phosphate aggregates are also associated with nanoparticles, and the nacreous layer is already visible immediately beneath them.

Figure 7a also displays the carbon (C) map, which outlines the mantle layer. Spherical structures, possibly corresponding to mucus, can be observed. In addition, fibers forming the internal structure of the mantle are visible. Figure 7(b.1,b.2), obtained from another shell specimen, also show the presence of P, Ca, and C at the mantle–nacre interface.

Figure 7(c.1) depicts the inner surface of the mantle and a structure known as the mantle rim [43]. Due to tissue dehydration, the mantle edge appears displaced toward the center of the shell. Detailed images of torn mantle areas (outlined in red in Figure 7(c.1)) reveal the inner surface of the mantle in contact with the shell. Region A4 (Figure 1b) is one such area where the torn mantle allows investigation of the surface in contact with the *L. fortunei* shell. The elemental maps in Figure 7(c.2) show the distribution of carbon (C) in the internal mantle fibers, mucus spheres, and other carbon-containing structures. In addition, phosphorus (P) and calcium (Ca) are distributed throughout the inner surface of the mantle in contact with the shell.

### 3.6. Region B: Mantle Edge and Mantle Surface

**Figure 8:**
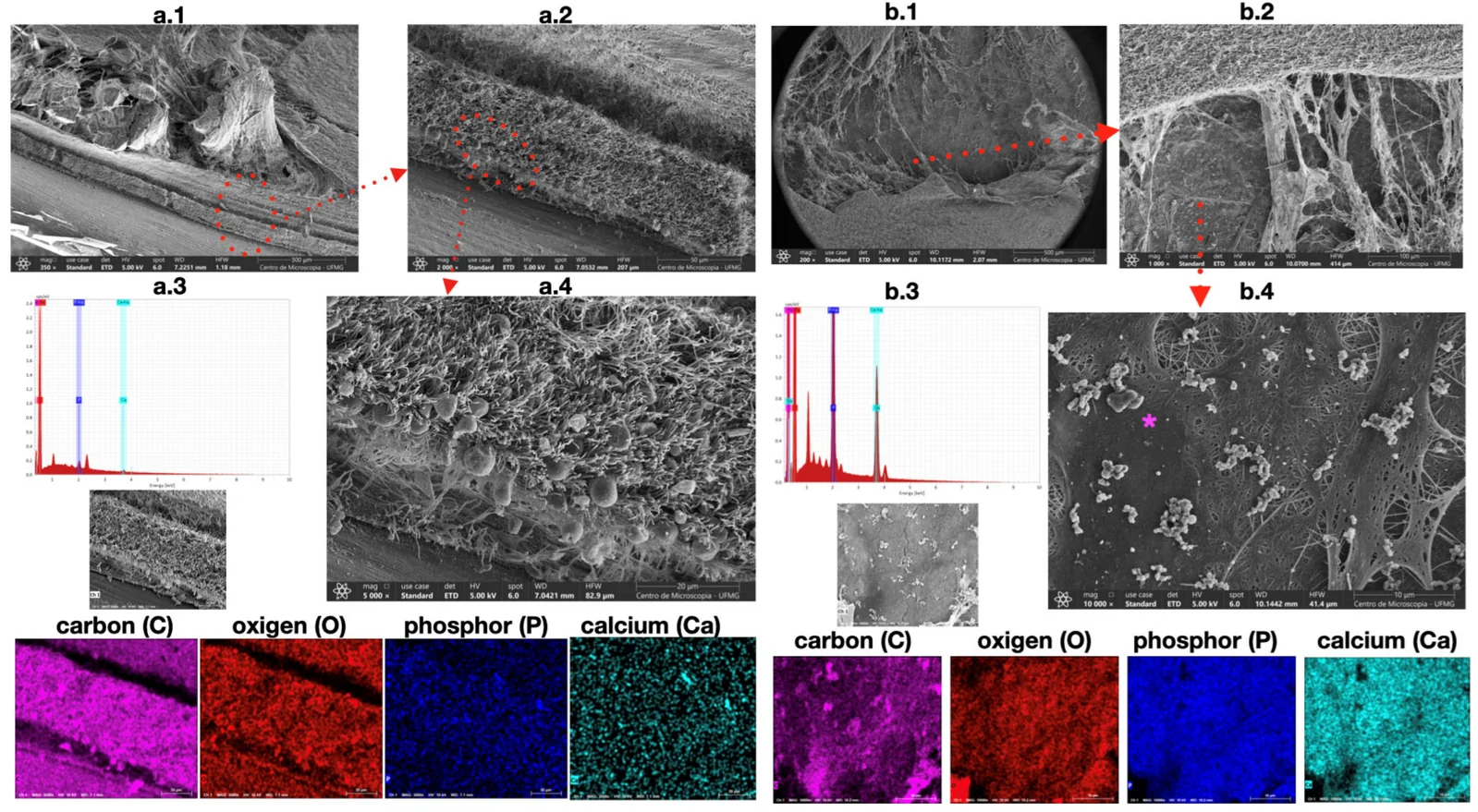
Scanning electron microscopy (SEM) images of the mantle folds are shown in panels (**a.1**,**a.2**,**a.4**). The folds are regions with a high density of cilia, as shown in (**a.2**). Panel (**a.4**) shows numerous mucus spheres produced by this fold. The EDS analysis in (**a.3**) and the corresponding elemental maps indicate that carbon (C) and oxygen (O) outline the region, whereas the concentrations of phosphorus (P) and calcium (Ca) are very low. SEM images of the mantle surface and interior are shown in (**b.1**,**b.2**,**b.4**). The ciliated surface of the mantle is clearly visible in b.1 and is better visualized in (**b.2**). The large number of cilia present on the surface is believed to aid in capturing micro- and nanometer-sized particles from the water. Image (**b.4**) shows the inner surface of the outer mantle layer, which is in contact with the shell. Several layers of fibers and particle aggregates (magenta asterisk) are visible on this surface. The EDS analysis in (**b.3**) and the corresponding elemental maps indicate that the particle aggregates on the inner surface of the outer mantle layer are rich in carbon. The concentrations of phosphorus (P), oxygen (O), and calcium (Ca) are significantly higher than those observed in the spectrum shown in (**a.3**).

The mantle folds are essential structures for understanding the biomineralization process. The process begins with the secretion of a film called the periostracum, which is composed of chitin, collagen, and proteins [45]. This film is secreted by the outer mantle fold and is formed by two adherent layers. Understanding this process is essential for understanding biomineralization. The secretion by the cells located in the outer fold, its polymerization into a hydrophobic film, and its adhesion to the pre-existing periostracum are still not well understood. Two additional folds, namely the middle and inner folds, have also been identified. These structures are visible in the SEM images of the mantle folds (Figure 8(a.1,a.2,a.4)). Figure 8(a.1) reveals that the folds are regions with a high density of cilia, which are better visualized in Figure 8(a.2). In addition, Figure 8(a.4) shows the large number of mucus spheres produced in this fold. The EDS analysis in Figure 8(a.3) includes elemental maps showing that carbon (C) and oxygen (O) outline the entire ciliated region. Carbon also outlines the mucus spheres. However, the presence of phosphorus (P) and calcium (Ca) in the EDS spectrum shown in Figure 8(a.3) does not allow specific identification of any structure because of their low concentrations.

SEM images, Figure 8(b.1,b.2,b.4) show the inner surface of the mantle, which encloses the viscera of *L. fortunei*. The ciliated surface is visible in Figure 8(b.1) and is better visualized in Figure 8(b.2). The numerous cilia are likely essential for capturing micro- and nanometer-sized particles when the shell is open. Figure S4 in the Supplementary Material shows that the mantle of *L. fortunei* has ciliated regions surrounding non-ciliated regions where particles may interact with the mantle surface.

In addition, Figure 8(b.4) shows the inner surface of the outer mantle layer, which is in contact with the shell. This surface exhibits several layers of fibers and particle aggregates. The EDS analysis in Figure 8(b.3) and the corresponding elemental maps indicate that the particle aggregates on the inner surface of the outer mantle layer are rich in carbon (C). The concentrations of phosphorus (P), oxygen (O), and calcium (Ca) are significantly higher than those observed in the spectrum shown in Figure 8(a.3).

### 3.7. Quantification of the Chemical Composition of Shell Edge Regions by EDS and Ca/P Ratio

In the shell-edge fractures shown in the previous figures, where distinct morphologies consistent with phosphate compounds were identified, the elements carbon (C), oxygen (O), phosphorus (P), and calcium (Ca) were quantified by EDS. Table 1 presents these quantifications for the typical microstructures of shell-edge regions A1, A2, and A3 of *L. fortunei*, previously shown in Figure 1 and the subsequent figures. The images are from two *L. fortunei* individuals, designated C1 and C2 for simplicity. Rows 1–3 of the table correspond to shell-edge region A1 of individual C1. Rows 4 and 5 correspond to shell sample C2. Row 6 shows the results for region A2 of shell sample C1, and row 7 corresponds to region A3 of the shell edge of sample C1. The images shown in the last column of the table correspond to the areas where the EDS quantifications were performed.

The Ca/P ratios (atomic ratios) were calculated from the atomic percentages obtained by EDS (rows 1–7 of Table 1). For comparison, the theoretical Ca/P molar ratios of hydroxyapatite (HA, 1.67) and carbonated hydroxyapatite (1.8) are shown at the bottom of the table. Some Ca/P atomic ratios obtained by EDS (rows 2 and 4) are close to the theoretical HA value (1.67), whereas the remaining values differ considerably from those of both HA and carbonated HA. The highest Ca/P ratio was observed in region A2, hypothetically owing to a more advanced stage of phosphate-to-carbonate substitution. Quantifications of magnesium (Mg) and sodium (Na) are also included to show that these elements are consistently present at much lower concentrations. Nitrogen (N) was included in the table for some morphologies to reflect their origin, as was zinc (Zn), which is shown in Figure S7 of the Supplementary Material.

### 3.8. Quantification of the Chemical Composition of L. fortunei and P. perna Shells by ICP-OES

Table 2 shows the concentrations of various metals and non-metals in the shells of the freshwater golden mussel (*Limnoperna fortunei*). The concentration of calcium is very high because it is the main component of the shell. Ba, Fe, Mg, Sr, Na, and Zn are present at concentrations of fractions of a percent. These metals, together with traces of other heavy metals present in both freshwater and seawater, were assimilated through the mantle and gills or absorbed by the digestive system. Sodium (Na) reaches concentrations in the tenths-of-a-percent range, probably because it is essential for most metabolic processes. The concentrations of the other metallic elements are very close to those reported in previous studies [46,47], especially for calcium, since CaCO_3_ represents more than 95% of the shell composition.

The concentration of phosphorus (P) is in the ppm range throughout the shell and differs greatly from the values found at the shell growth edge shown in Table 1. Studies reporting the chemical composition of the shell generally do not report the presence of phosphorus, possibly because it occurs at very low concentrations [48].

### 3.9. Quantification of the Chemical Composition of L. fortunei and P. perna Shells by WDXRF

Table 3 shows the concentrations of different metals and non-metals present in the shells of *L. fortunei* (freshwater) and *P. perna* (marine), as determined by wavelength-dispersive X-ray fluorescence (WDXRF). These values are semiquantitative but are in excellent agreement with the concentrations obtained by ICP-OES. Because the samples consisted of whole shells, the phosphorus concentrations are in the ppm (parts per million) range. Therefore, the occurrence of calcium phosphate is likely restricted to the shell growth edge, both in area and thickness. The calcium concentration exceeds 98% in the shells of both the freshwater bivalve *L. fortunei* and the marine bivalve *P. perna*. The concentrations of all other chemical elements are below 2%.

### 3.10. X-Ray Diffraction (XRD) of the Shell Tips of L fortunei and P perna

**Figure 9:**
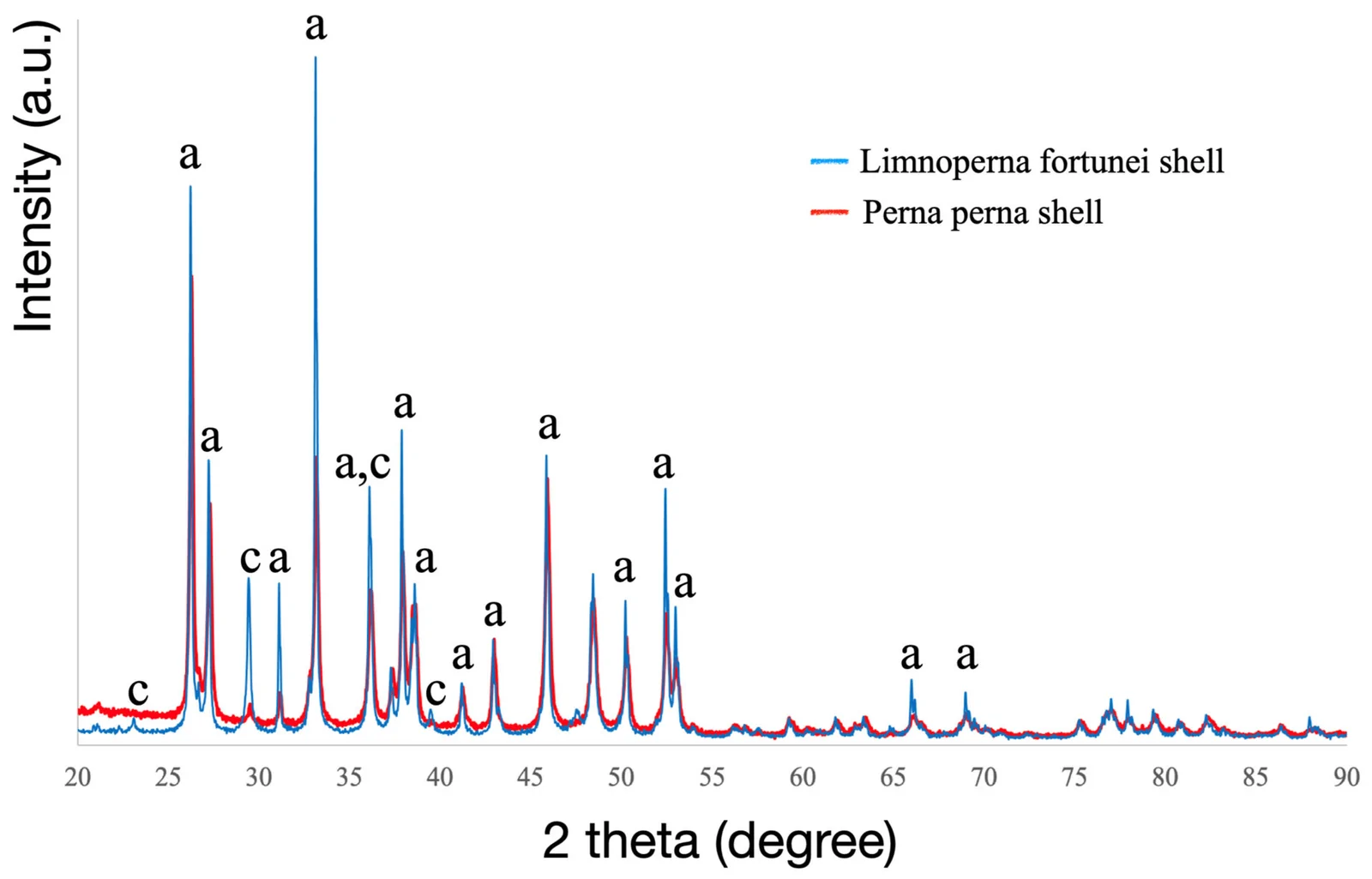
X-ray diffraction patterns of mussel shells. The XRD patterns of *L. fortunei* and *P. perna* show the presence of aragonite and calcite. No calcium phosphate phases were detected. a: aragonite; c: calcite.

Figure 9 shows the X-ray diffraction (XRD) patterns of the shells of *L. fortunei* and *P. perna*. As can be seen, the diffraction patterns are practically identical, indicating the presence of the aragonite and calcite phases. Aragonite is the predominant phase in both shells. The diffraction patterns indicate an approximate aragonite-to-calcite peak height ratio of 10:1, based on the peaks located at 2θ = 46° for aragonite and 2θ = 40° for calcite [49].

No other crystalline phase was detected, in agreement with the ICP-OES and WDXRF results, which indicate a very low phosphorus (P) concentration in the shell. This makes the detection of apatite, hydroxyapatite, or other phosphorus-containing phases by XRD difficult. However, we could not identify the diffraction peak at 2θ = 26.21°, which may correspond to one of the main diffraction peaks of hydroxyapatite [25.9° (002)] [50]. It should also be noted that the diffraction peaks of calcium phosphate crystalline phases, especially hydroxyapatite, largely overlap with those of aragonite and calcite, making their detection by XRD difficult.

### 3.11. FTIR and ATR-FTIR Spectroscopy of the Shell Tips of L fortunei and P perna

**Figure 10:**
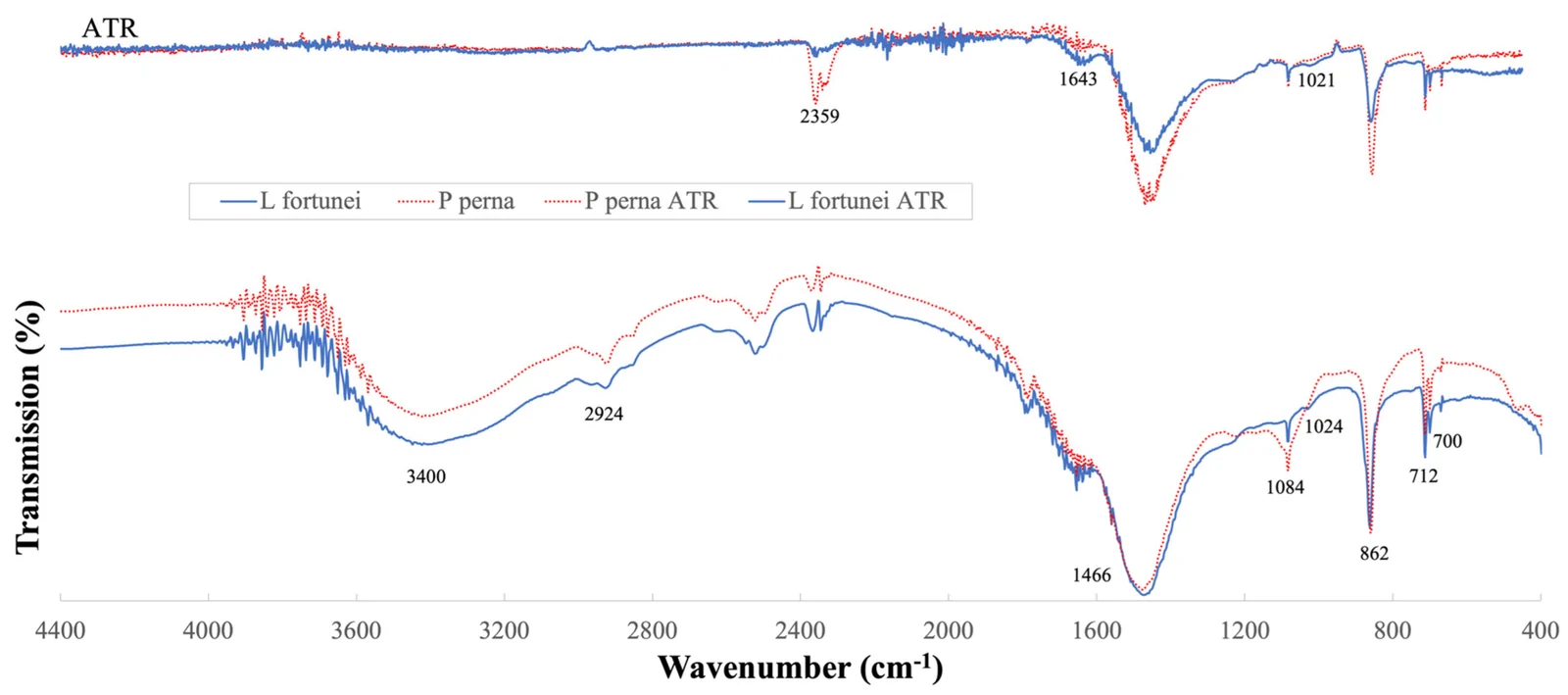
FTIR and ATR-FTIR spectra (upper part of the figure) of mussel shells. The aragonite phase is identified in all FTIR and ATR-FTIR spectra of *L. fortunei* and *P. perna* by the absorption bands at 700, 712, 862, 1084, and 1466 cm^−1^. The band at 2924 cm^−1^ may be attributed to chitin [52] present in the shell. The broad band between 3000 and 3600 cm^−1^ is attributed to the stretching vibration of hydroxyl (OH) groups and to adsorbed water. This band is absent from the ATR-FTIR spectrum. The absorption bands at 1643 and 2359 cm^−1^, as well as other unassigned bands in the FTIR spectrum, can be attributed to carbonate species [53,54]. The absorption band at 1024 cm^−1^ in the FTIR spectra of *L. fortunei* and *P. perna* shells can be attributed to the phosphate group (PO_4_^3−^) [55,56].

Figure 10 shows the FTIR and ATR-FTIR spectra (upper part of the figure) obtained from shell tip samples of *L. fortunei* and *P. perna*, in which the main functional groups could be identified. Carbonate is observed in all FTIR and ATR-FTIR spectra of *L. fortunei* and *P. perna*. It is characterized by absorption bands at 700, 712, 862, 1084, and 1466 cm^−1^ [51]. The band at 2924 cm^−1^ is probably associated with the presence of chitin in the shell and in the attached periostracum [52]. The bands between 3000 and 3600 cm^−1^ are attributed to hydroxyl groups and adsorbed water. These features were not observed in the ATR-FTIR spectra. The absorption bands at 2359 and 1643 cm^−1^ may be attributed to the asymmetric stretching of atmospheric CO_2_ [53,54].

The phosphate group is observed as very weak and broad absorption bands at 1024 cm^−1^ (FTIR) and 1021 cm^−1^ (ATR-FTIR) in the shells of *L. fortunei* and *P. perna*. [55,56]. Other absorption bands attributed to the phosphate group may partially or completely overlap with those associated with the carbonate group and therefore could not be identified with certainty.

## 4. Discussion

The SEM images acquired using backscattered electron (BSE) imaging and energy-dispersive X-ray spectroscopy (EDS) for elemental mapping of calcium (Ca), carbon (C), phosphorus (P), and oxygen (O), shown in Figure 2, Figure 3, Figure 4, Figure 5, Figure 6, Figure 7 and Figure 8, together with the concentrations of these elements presented in Table 1 for selected areas of the shell edge of *L. fortunei*, indicate that calcium phosphate occurs within the mantle (Figure 7 and Figure 8) and on both the inner and outer surfaces of the pallial epithelium of the *L. fortunei* mantle. We did not observe any overlap between carbon and calcium either within the mantle or on its outer surface. Calcium carbonate (CaCO_3_), in contrast, is clearly observed in the nacreous layer (Figure 7). In Figure 7(a.1–a.3), calcium phosphate, identified by the phosphorus and calcium elemental maps, forms a continuous film approximately 3 μm thick that separates the epithelial surface of the mantle from the nacreous layer. In Figure 7(a.3), this phosphorus- and calcium-containing film is composed of particles that appear to be undergoing dissolution and subsequently self-organize into the plate-like structures of the underlying nacreous layer.

Tears in the inner surface of the mantle of *L. fortunei*, shown in Figure 8(b.1,b.2,b.4), reveal that the inner surface of the mantle is ciliated and that the mantle interior has a complex fibrous structure. The carbon (C) map highlights organic fibers and microscopic aggregates (one of them marked with a magenta asterisk), possibly consisting of mucus, within the mantle. Note that neither calcium nor phosphorus marks these structures (compare with the secretions produced by the cilia of the inner mantle fold in Figure 8(a.4)). It is not possible to conclude that this mucus originated within the mantle, as it may have been introduced during sample preparation (see Figure 1 for an overview of the areas examined).

The images of the shell edge confirm and describe in greater detail the growth process of the *L. fortunei* shell. Figure 2 shows images of the growth margin at the shell tip opposite the umbo. Damage to the periostracum at this location made it possible to observe the biomineralization process immediately beneath it (Figure 2(a.1). The Ca, C, P, and O elemental maps show that calcium and phosphorus are associated with the morphologies shown in Figure 2(a.2,a.3) (surface topography) and Figure 2(c.1,c.3) (images with greater depth of field). The striking similarity of these morphologies to those of hydroxyapatite (HAp) can be observed in studies in which HAp exhibits a plate-like morphology [57,58]. The Ca/P ratio is used to characterize the type of calcium phosphate compound, and the value of 1.66 obtained by EDS coincides with the theoretical Ca/P ratio of HAp, as shown in Table 1 [59].

The images in Figure 3a,b are higher-magnification views of the plate-like morphology. The growth of these plates may occur through the adhesion of nanoparticles, which are present in large numbers in the images. The images in Figure 3c,d, obtained from another *L. fortunei* shell specimen, confirm the presence of plate-like calcium phosphate-containing composites at the shell growth edge (see the EDS elemental maps in Figure 3e). The thickness of the HAp layer in Figure 3d is approximately 18 μm, whereas the total thickness of the calcium phosphate layer is approximately 120 μm. Note in the detail of Figure 3d that a surface appears to form over the first HAp layer (marked with a red asterisk). This type of structure may contribute to the mechanical stiffness of the growing shell tip.

One question that remains is whether this calcium phosphate layer, whether composed of HAp, carbonated hydroxyapatite (CHAp), or another phosphate phase, remains stable or is gradually replaced by CaCO_3_ as the shell grows. The main challenge is that the phosphorus concentration in the shell is well below 1%, as determined by ICP-OES and WDXRF, and is therefore below the detection limit of X-ray diffraction (XRD). This limitation was the primary reason for conducting a detailed investigation of the shell growth front at the micrometer scale using SEM and EDS.

The SEM images, elemental maps, and EDS compositional spectra shown in Figure 4 are from region A2 of the *L. fortunei* shell (Figure 1b). This region is farther from the growing shell tip. Once again, fractures in the periostracum made it possible to analyze the composition and morphology of the calcium phosphate aggregates that occur at the shell edge, precisely where the periostracum curves and joins the mantle edge from which it is secreted. The aggregates do not exhibit the same morphology as those shown in Figure 2(a.3). Details of this morphology are presented in Figure 5(a.1) and show that, beneath the calcium phosphate aggregates, a layer of plates (Figure 5(a.2,a.3)) is formed that closely resembles the CaCO_3_ nacreous layer of the *L. fortunei* shell. The nanoparticles circled in red in Figure 5(a.3) may be incorporated into the nacreous layer during its formation. The void that allowed the nacreous layer to be observed may have resulted from the dissolution of the micrometer-sized particles shown in Figure 5(b.1), which have a large surface area characterized by numerous indentations and may react with substances secreted by the mantle surface, which also secretes calcium phosphate.

Miyashita et al. [60] showed that nacrein inhibits the growth of calcium carbonate. In this work, we show that enzymes dissolve calcium phosphate at the edge of the *L. fortunei* shell and that calcium carbonate is produced through this dissolution–precipitation process. Biomineralization of the *L. fortunei* shell is therefore a source of phosphorus compounds released into the organism as the shell grows.

In *L. fortunei*, the transformation occurring at the shell edge (calcite), which promotes shell growth in area, differs from that occurring through the shell thickness (aragonite). At the shell edge, a morphology identical to that of HAp undergoes transformation. This morphology undergoes dissolution, and continuous surfaces perpendicular to the plates develop through dissolution of the phosphate phase, giving rise to the cubic morphology of calcite. A proposed mechanism for this process is shown in Figure S5 of the Supplementary Material.

The SEM images and compositional maps shown in Figure 6 are from region A3 of the *L. fortunei* shell (Figure 1b). In this case, two fractures are present at the shell edge, immediately beneath the periostracum. In Figure 6(a.1–a.3), the presence of a phosphorus- and calcium-containing layer can be observed, although its morphology could not be characterized. However, Figure 6(b.1–b.4) clearly show that the particle morphology is the same as that observed in Figure 3. Analysis of these images indicates that the plate-like morphologies undergo a coalescence process. This coalescence may result from the action of substances with dissociative capacity.

Our experiments on the shell of *L. fortunei* confirm that calcium phosphate is located at the growing shell edge and, consequently, adjacent to the periostracum. At the beginning of shell growth, the mantle—a structure larger than the shell itself—must first produce periostracum films (comprising at least three layers formed within the mantle groove [61,62] over the existing shell. These films must firmly adhere to the pre-existing periostracum before the biomineralization process can begin. In *L. fortunei*, the distances between the growth lines visible on the shell surface range from submillimeter to several millimeters. In the marine bivalve *P. perna*, the growth lines are separated by several millimeters because its shell is much larger than that of *L. fortunei*.

**Figure 11:**
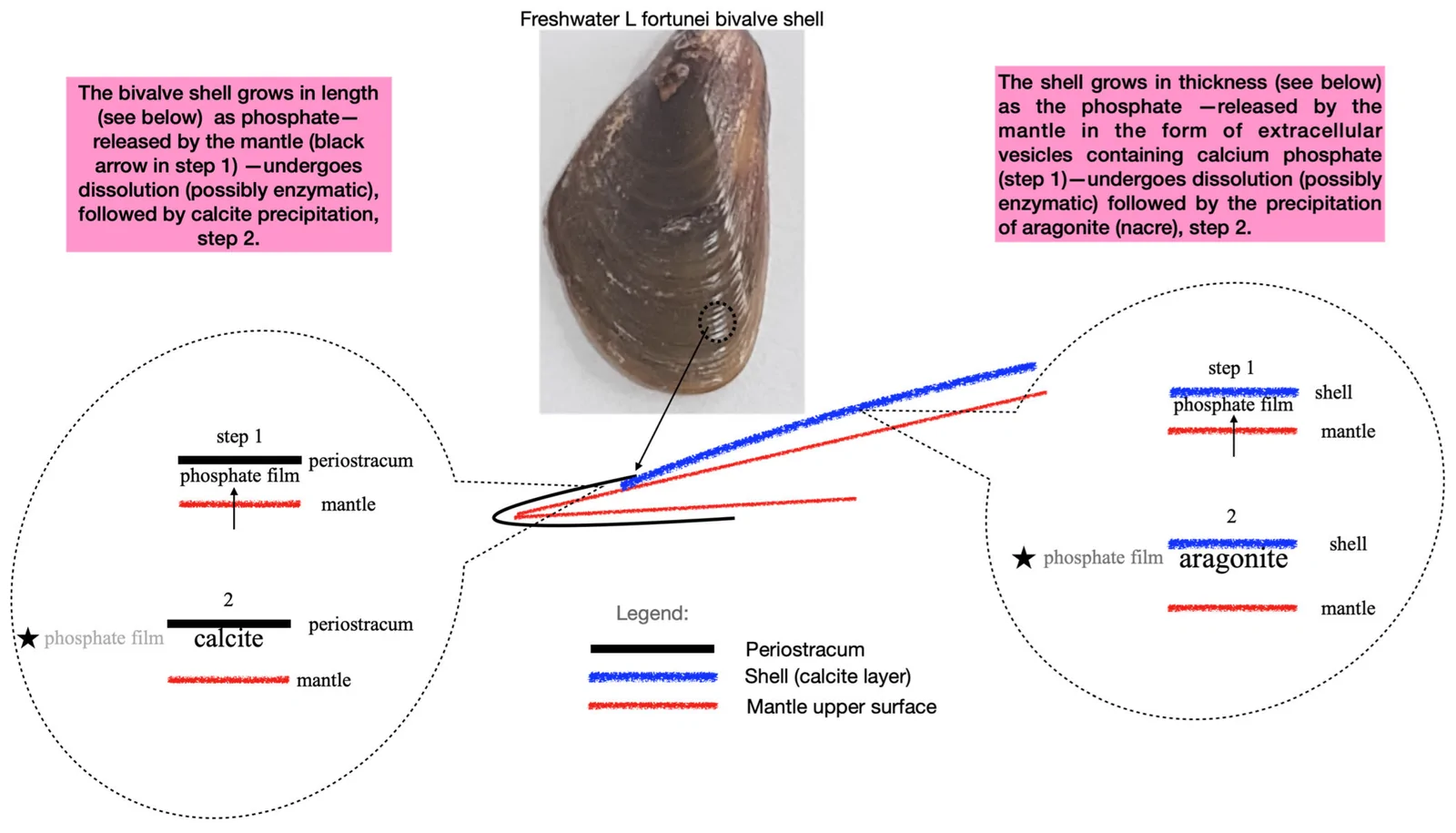
Proposed diagram of the stages of shell growth in *L. fortunei*. Periostracum sheets (black line) are secreted by the outer mantle fold (red line) and adhere to the pre-existing shell surface (blue line). The distances between successive growth fronts are in the millimeter to submillimeter range in *L. fortunei*. The dashed circle in the shell photograph highlights the biomineralized protrusion formed at the overlap between the newly deposited periostracum and the pre-existing shell. On the left side of the diagram, shell growth in length is illustrated. XRD and FTIR results from the present and previous studies [35,67] indicate the presence of calcite adjacent to the periostracum. The periostracum remains connected to the mantle at the shell edge (see the mantle edges in Figure 1). The star (★) followed by the gray label “phosphate film” represents the action of substances (carbonic anhydrase or other enzymes) secreted by the mantle that may promote phosphate-to-carbonate replacement. On the right side of the diagram, shell growth in thickness is illustrated. The action of organic substances (★) transforms the nanoparticles that constitute the calcium phosphate film shown in Figure 7 into aragonite plates.

A schematic diagram of *L. fortunei* shell growth is shown in Figure 11. Thin hydrophobic periostracum layers, already polymerized, are secreted by the outer mantle fold and adhere to the existing shell surface. In Figure 11, the dashed circle around the photograph of the *L. fortunei* shell highlights the biomineralized ridge formed at the overlap between the newly deposited periostracum and the pre-existing shell. On the right side of the diagram, the increase in shell thickness is illustrated. The action of substances (marked with ★) transforms the nanoparticles that constitute the calcium phosphate film shown in Figure 7 into aragonite plates.

What are the possible competitive advantages of CaCO_3_ replacement from a calcium phosphate precursor? The first, and perhaps the most important, is that phosphate may be acquired primarily directly from the water, accumulated at the mantle edges [21], and subsequently transferred to the pallial surface of the mantle in the form of nanoparticles or microparticles. This transformation would provide a source of phosphoric acid species [63], which are essential for energy production (ATP), cell repair, pH regulation, and electric impulses. The mechanism of bone resorption may have its genetic ancestor in this stage of mantle biomineralization [63].

Another advantage of the presence of crystalline calcium phosphate phases at the shell tip (Figure 2, Figure 3 and Figure 6) would be to provide greater rigidity to the system during biomineralization. The presence of amorphous phases would not provide the compressive strength exhibited by HAp [64], which may be necessary to maintain the mechanical rigidity required for continued biomineralization. The compressive strength of calcite and aragonite is lower than that of HAp.

The presence of a thin layer of crystalline HAp on the inner cuticle of bird eggs has been known for decades [65]. The morphology of these HAp crystals also resembles that of the crystals observed here beneath the periostracum of *L. fortunei*.

In bird eggs, the HAp layer is the last layer deposited (from the inside to the outside of the shell) and has been proposed to terminate calcite growth. However, subsequent work [66] showed that phosphorus, although present at very low concentrations, occurs throughout the eggshell thickness. Could this phosphorus be evidence of a phosphate-to-carbonate replacement process, with the thin HAp layer on the eggshell cuticle marking the end of the biomineralization cycle?

In *L. fortunei*, the calcium phosphate layer is the first to be deposited after being secreted by the mantle. Rather than blocking CaCO_3_ growth, the phosphate phase dissolves while carbonate precipitates. This replacement may occur because the bivalve secretes substances such as carbonic anhydrase and related enzymes that promote this process. At the shell tip (shell growth in length), crystalline HAp is replaced by crystalline carbonate phases, either calcite or aragonite. During shell thickening (nacre formation), the phosphate film may be amorphous and transform into amorphous calcium carbonate (ACC) nanoparticles, which subsequently aggregate and transform into plate-like aragonite (a metastable phase).

## 5. Conclusions

At the shell edge of the bivalve *L. fortunei*, calcium phosphate phases are the first mineral phases to be deposited beneath the periostracum, both during shell growth in length and during shell thickening through the formation of the nacreous layer. In *L. fortunei*, this process occurs on the micrometer scale, with the phosphate film on the epithelial surface of the mantle being approximately 3 μm thick.

At the growing shell tip (Figure 2 and Figure 3), calcium phosphate compounds with morphologies closely resembling the crystalline hydroxyapatite (HAp) phase were identified as the first mineral phase deposited beneath the periostracum. The HAp plates, or similar calcium phosphate compounds, tend to coalesce and form continuous surfaces (red asterisks in Figure 2(c.2), Figure 3d, and Figure 6(b.4)). The presence of crystalline phases may contribute to the mechanical stiffening of the growing shell edge. A proposed mechanism for the transformation into calcite is presented in Figure S5 (Supplementary Material).

In the region of the *L. fortunei* shell that grows in thickness, a calcium phosphate film composed of nanoparticles was observed (Figure 7a and Figure S6 in the Supplementary Material). Immediately beneath this film lies the nacreous layer, composed of the characteristic aragonite tablets (or bricks). A clear separation exists between the epithelial surface of the mantle, the calcium phosphate nanoparticle film, and the nacreous layer. Images from the lateral shell edge (Figure 4 and Figure 5) show aggregates identified as calcium phosphate adjacent to the periostracum and, immediately beneath them, the formation of tablets (or bricks) labeled by calcium (Ca) but no longer by phosphorus (Figure 4(b.1)).

The chemical composition of the shells of the freshwater bivalve *L. fortunei* and the marine bivalve *P. perna*, determined by ICP-OES and WDXRF, indicates that phosphorus (P) is present at ppm concentrations, demonstrating that calcium phosphate phases in the shell are restricted to micrometer-scale films undergoing replacement by calcium carbonate.

The phosphate absorption band around 1020 cm^−1^ was tentatively assigned in both FTIR and ATR-FTIR spectra. In contrast, carbonate exhibited well-defined absorption bands.

The presence of phosphate as the primary phase in the biomineralization process of *L. fortunei* reopens the discussion on the importance of phosphate-to-carbonate replacement during bivalve biomineralization and suggests an important evolutionary advantage through the release of phosphate compounds essential for energy production and other metabolic functions of the organism.

## Figures and Tables

**Table 1:** Elemental composition (weight %, semiquantitative) of regions A1, A2, and A3 at the shell edge of *L. fortunei* (images in the last column), obtained by EDS analysis, together with the Ca/P ratios calculated from weight % and atomic %. The lower part of the table shows the chemical composition, Ca/P ratios calculated from weight % and atomic %, of carbonated hydroxyapatite from bone, as proposed by LeGeros [44] (marked with a black asterisk) and the theoretical molar ratio of hydroxyapatite.

Line		Elements	EDS Quantification—Composition (Weight %)					EDS Quant.-(Atom %)		Ratio Ca/P	Other Elements			Image of the Region
	Shell Region		C	O	P	Ca	Ca/P	P	Ca		N	Mg	Na	
**1**	C1-A1		10.0	-	7.2	25.5	3.5	13.7	48.9	3.57	-	-	-	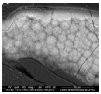
**2**	C1-A1		37.0	23.6	13.3	28.6	2.2	7.5	12.4	1.66	-	0.2	1.2	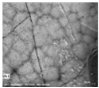
**3**	C1-A1		58.5	26.9	3.7	12.6	3.4	1.6	4.1	2.64	8.8	-	-	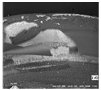
**4**	C2-A1		13.8	36.6	15.5	33.1	2.1	10.4	17.2	1.65	-	-	0.9	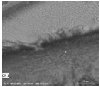
**5**	C2-A1		50.4	22.3	5.5	7.5	1.4	2.7	2.8	1.06	8.5	-	-	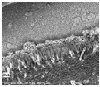
**6**	C1-A2		18.9	36.4	6.6	36.4	5.5	4.3	18.3	4.27	-	-	-	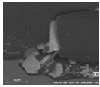
**7**	C1-A3		90.1	-	2.2	7.7	3.5	0.9	2.5	2.74	-	-	-	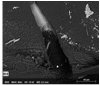
										Molar ratio Ca/P				
	Hydroxyapatite (HA)			Ca_10_(PO_4_)_6_(OH)_2_				10	6	1.67	-	-	-	
			Composition (weight %)											
					P	Ca	Ca/P				N	Mg	Na	
	Bone *		CO3^−2^ = 6		11.5	24.5	2.13	-	-	1.8	-	0.6	0.7	

**Table 2:** Elemental composition of metals and non-metals in the shells of *Limnoperna fortunei* and *Perna perna*, determined by ICP-OES (wt.%).

Sample (Shell)	Year	Ba	Ca	Fe	P	Mg	Na	Sr	Zn	K
*Limnoperna fortunei*	2016	0.0159	35.08	0.040	-	0.0235	0.220	0.059	0.0090	0.0032
*Limnoperna fortunei*	2022	0.0161	36.07	0.011	-	0.0180	0.537	0.120	0.0003	0.0037
*Limnoperna fortunei*	2023	0.0002	38.71	0.002	0.026	0.0190	0.640	0.120	0.0003	0.0190
*Perna perna*	2022	0.0003	38.64	0.002	-	0.0159	0.663	0.105	-	0.0133
*Perna perna*	2023	0.0340	39.05	0.043	0.039	0.0230	0.250	0.071	0.0008	0.0029

**Table 3:** Elemental composition of metals and non-metals in the shells of *Limnoperna fortunei* and *Perna perna*, determined by wavelength-dispersive X-ray fluorescence (WDXRF) (wt.%).

Sample (Shell)	Year	Al	Ba	Ca	Cl	Fe	K	Mg	Na	P	S	Si	Sr
*Limnoperna fortunei*	2023	0.051	-	98.190	0.280	0.033	0.044	0.050	0.760	0.037	0.240	0.120	0.195
*Perna perna*	2023	0.110	0.490	98.100	0.420	0.230	-	0.062	0.430	0.089	0.210	0.140	0.120

## Data Availability

The data supporting the results of this article will be made available by the authors on request.

## Supplementary Materials

The following supporting information can be downloaded at https://www.mdpi.com/article/10.3390/ani16162488/s1, Figure S1: The upper part of the photo shows the two shells of the bivalve *Limnoperna fortunei*. The lower part of the photo shows a mantle (pallium in Latin) of the golden mussel. Each shell arises from the biomineralization produced by the mantle of the bivalve; Figure S2: Anatomy of *L. fortunei*, showing the parts that are relevant to this work; Figure S3: Internal surface (facing the gills) of the *L. fortunei* mantle at different magnifications: (a) Displays the internal surface and interior of the *L. fortunei* mantle; (b) Cilia patches are evident on the internal surface of the mantle; (c) Detail of the cilia, with some areas devoid of cilia visible; (d) Using a SEM-T1 detector, black circles that appear to be micrometer-sized pores are observed on the mantle’s surface; (e) Detailed view of these pores with a particle situated on one of them. Further studies will investigate the nature of these openings to determine whether they are artifacts from sample preparation or genuine pores in the mantle wall; Figure S4: (a) Broken *L. fortunei* shell displaying the entire cross-section of the shell. Periostracum circled in blue; (b) Periostracum of *L. fortunei* featuring its two layers and the calcite layer beneath it; (c) Detail of the calcite layer below the periostracum; (d) and (e) Detailed views of the microstructure of the calcite layer; Figure S5: Transformation of calcium phosphate -> calcite at the growth edge of the *L. fortunei* shell via enzimatic: (a) Calcium phosphate plates with morphology similar to hydroxyapatite (HAp) or carbonated HAp (CHA), dahllite [68], exhibit coalescence, possibly through dissolution of calcium phosphate and precipitation of calcium carbonate [63]. (b) Monocrystals or polycrystals of calcite present immediately below the periostracum of the *L. fortunei* shell. See location in the images of Figure 3. Above each image, diagrams appear (step 1 and step 2) suggesting the process of dissolution of calcium phosphate and precipitation of calcium carbonate in the calcite phase. Dissolution takes place by protonation of the carbonate group to form carbonic acid and protonation of the phosphate groups to form phosphoric acid; Figure S6: (a) Surface of the mantle (lower part of the image) and nacre in the upper part of the image; (b) Between the mantle and the nacre the phosphate film; (c) Image of the phosphate film in detail. (d) and (e) show the same region using different SEM sensors; (f) Maps of the four main chemical elements observed in the image: carbon (C), phosphorus (P), oxygen (O) and calcium (Ca); (g) Nanoparticles in the phosphate film. Figure S7: (a) Sample from the edge of the *L*. *fortunei* shell (SEM) was analyzed by EDS; (b) Area circled in red is shown in detail. (c) The main chemical elements found were C, Ca, P, O and Zn, as shown in the atomic concentration graph in (d). (e) The element maps show that Zn marks the region where calcium (Ca), phosphorus (P) and oxygen (O) occur. Carbon (C) does not coincide with the other elements. (f) Shows in detail the region circled in black. The morphology of the crystals in the upper part of this image is identical to that shown in Figure 2, Figure 3 and Figure 6 of the article. The presence of zinc (Zn), a metallic element in the enzyme carbonic anhydrase (CA), was not found in other samples, only at this edge; Table S1: Carbonic Anhydrase Assay.
