# Supplementary material for: Dissolution of Phosphate and Precipitation of Carbonate in the Biomineralization of the Bivalve Shell Limnoperna fortunei
Source: Animals (Basel). 2026 Aug 10;16(16):2488. doi: 10.3390/ani16162488 (PMC13508937; doi:10.3390/ani16162488)
Supplement: Supplementary file 1 [file animals-16-02488-s001.zip › animals-4423310-supplementary.pdf]

Supplementary Material of "Dissolution of Phosphate and Precipitation of Carbonate in the Biomineralization of the Bivalve Shell Limnoperna fortunei"

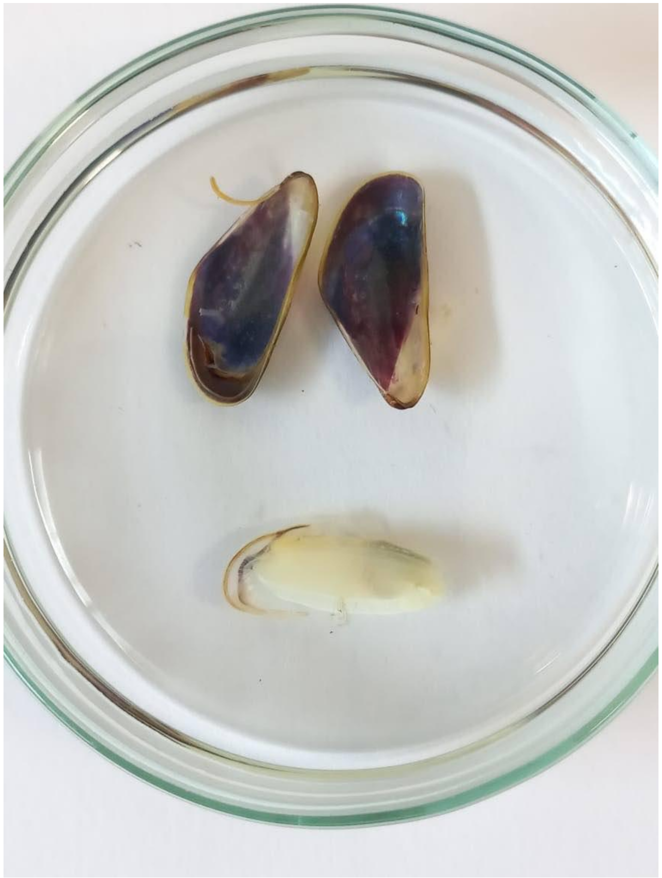

Figure S1- The upper part of the photo shows the two shells of the bivalve Limnoperna fortunei. The lower part of the photo shows a mantle (pallium in Latin) of the golden mussel. Each shell arises from the biomineralization produced by the mantle of the bivalve.

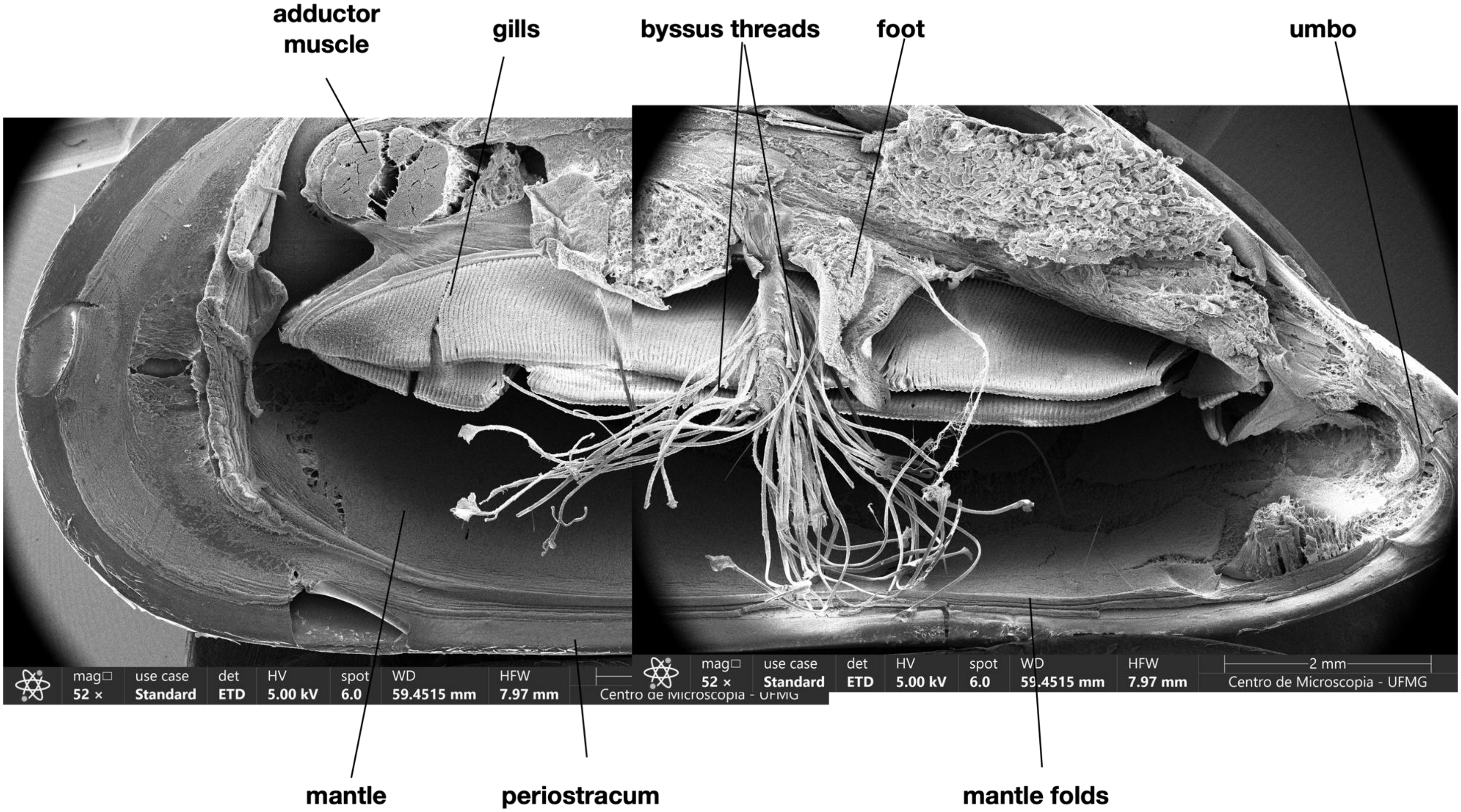

Figure S2 - Anatomy of L. fortunei, showing the parts that are relevant to this work.

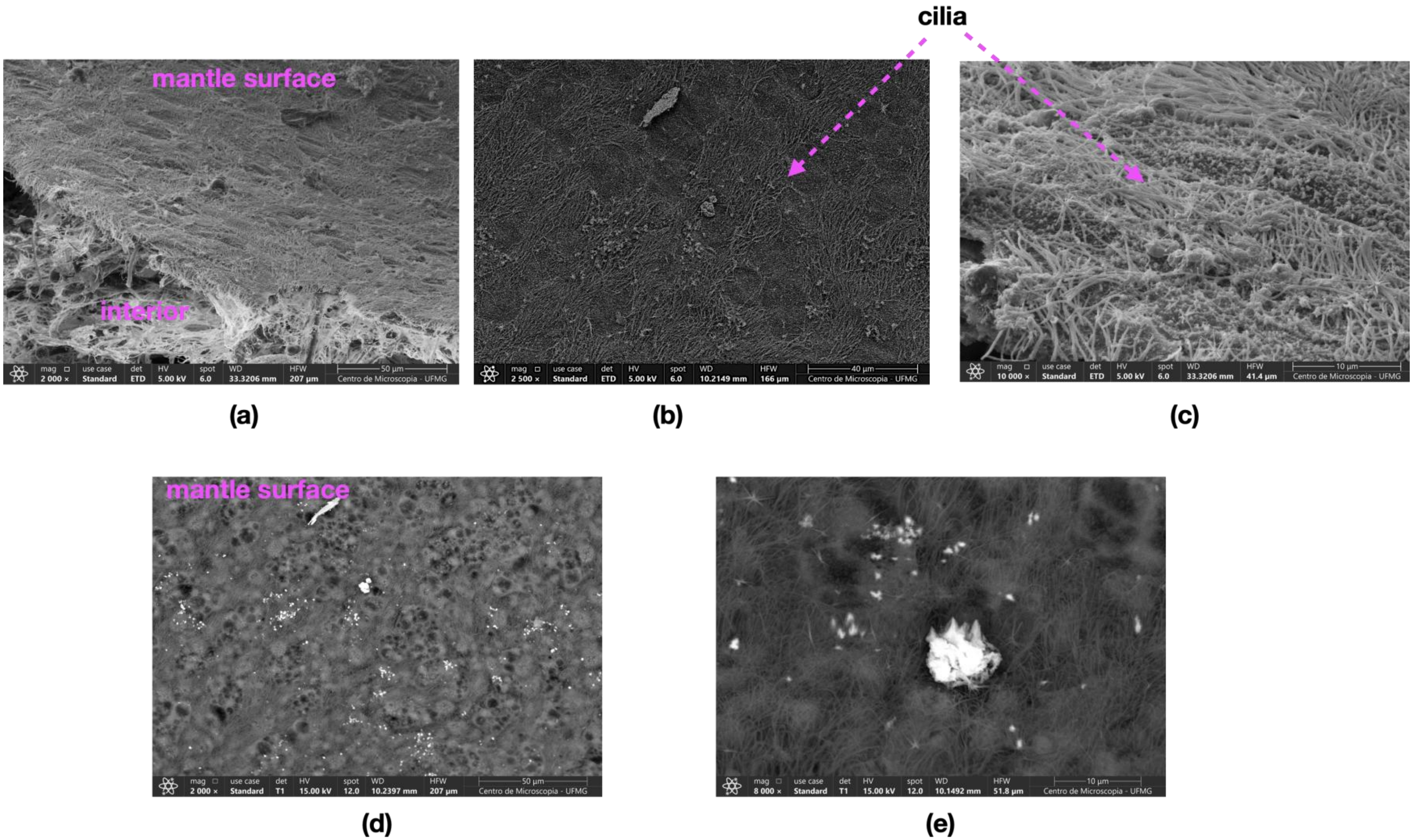

Figure S3 – Internal surface (facing the gills) of the L. fortunei mantle at different magnifications: (a) Displays the internal surface and interior of the L. fortunei mantle; (b) Cilia patches are evident on the internal surface of the mantle; (c) Detail of the cilia, with some areas devoid of cilia visible; (d) Using a SEM-T1 detector, black circles that appear to be micrometer-sized pores are observed on the mantle's surface; (e) Detailed view of these pores with a particle situated on one of them. Further studies will investigate the nature of these openings to determine whether they are artifacts from sample preparation or genuine pores in the mantle wall.

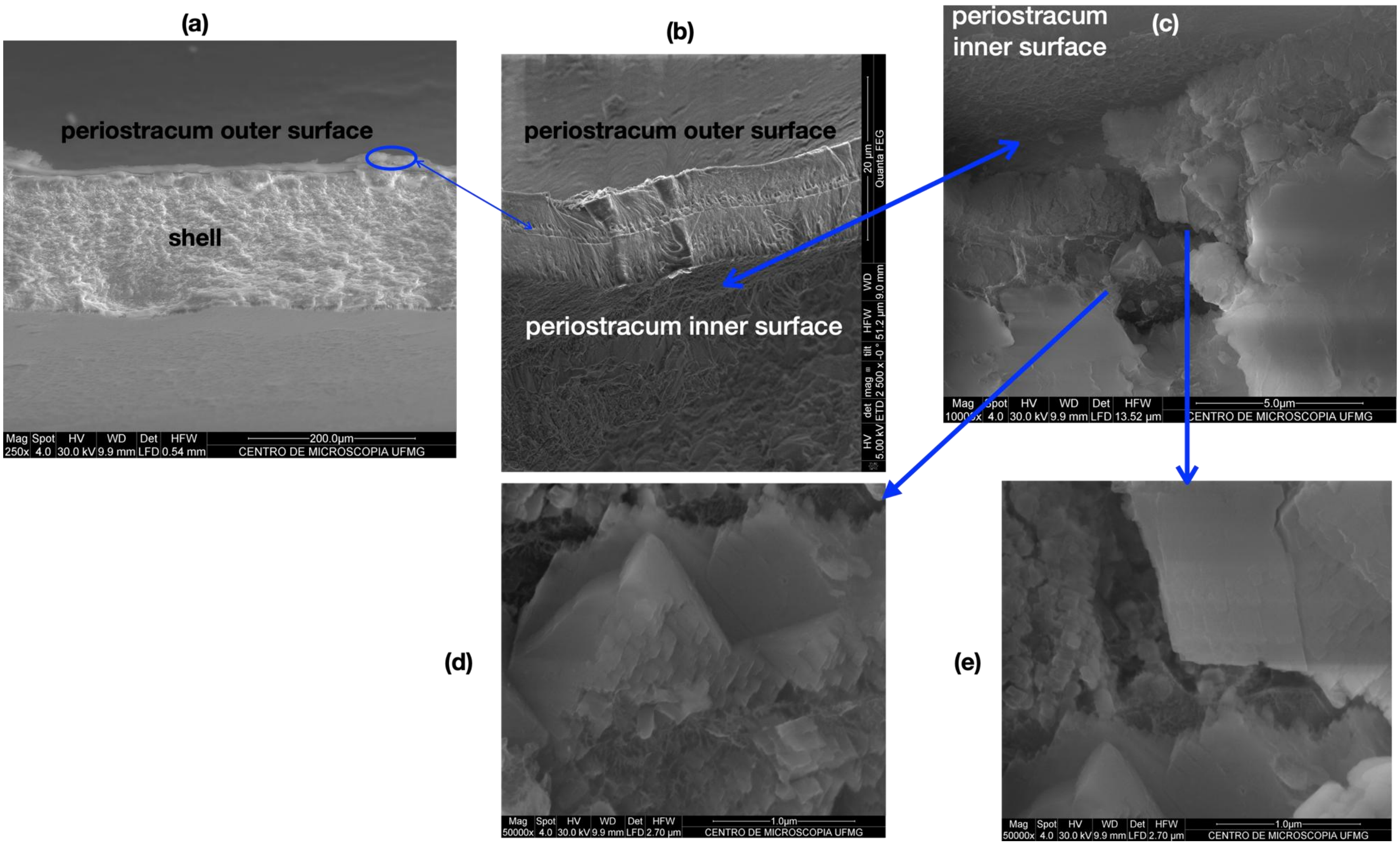

Figure S4 – (a) Broken L. fortunei shell displaying the entire cross-section of the shell. Periostracum circled in blue; (b) Periostracum of L. fortunei featuring its two layers and the calcite layer beneath it; (c) Detail of the calcite layer below the periostracum; (d) and (e) Detailed views of the microstructure of the calcite layer.

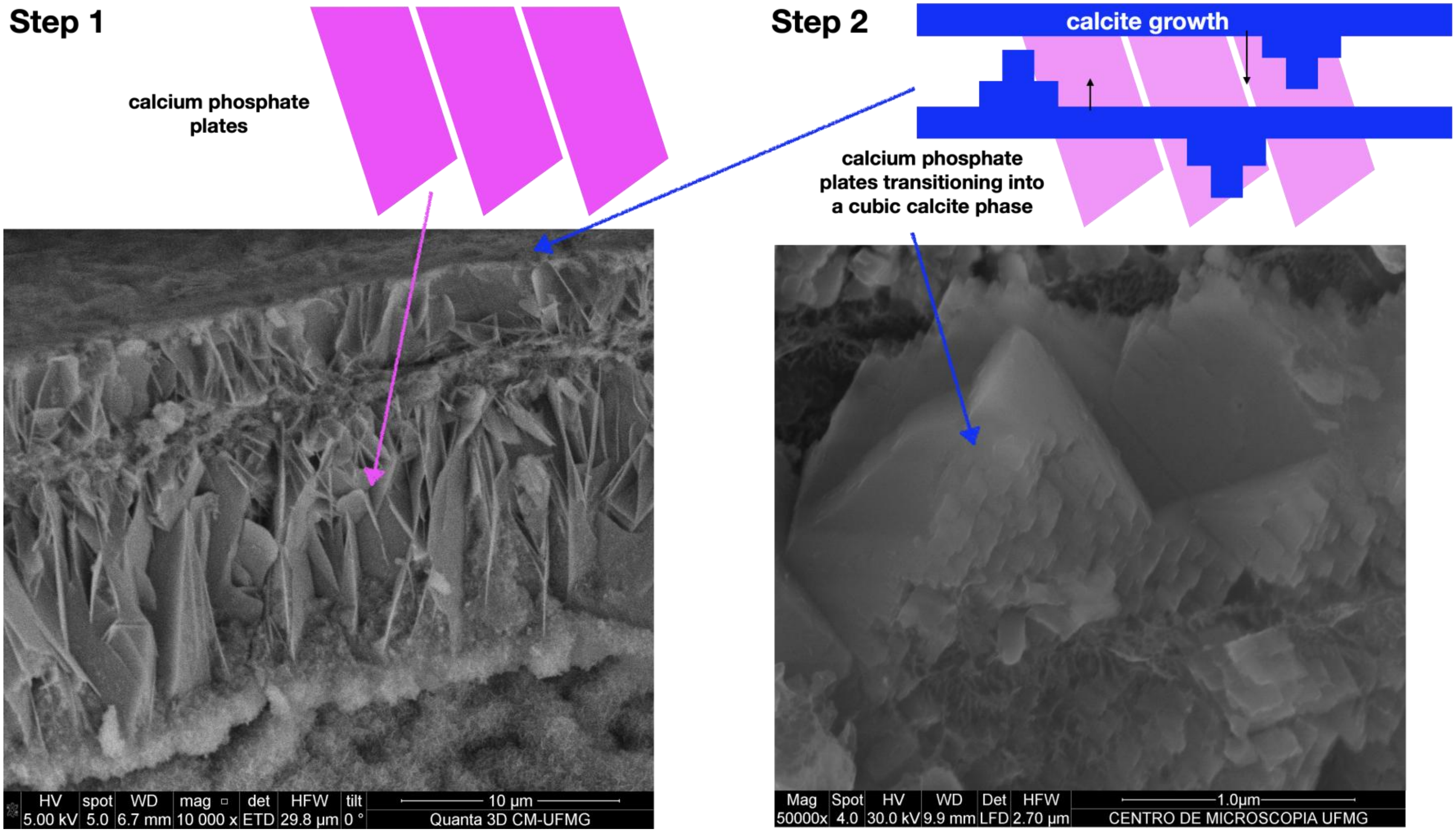

(a) (b)

Figure S5- Transformation of calcium phosphate -> calcite at the growth edge of the L. fortunei shell via enzimatic : (a) Calcium phosphate plates with morphology similar to hydroxyapatite (HAp) or carbonated HAp (CHA), dahllite (1) , exhibit coalescence, possibly through dissolution of calcium phosphate and precipitation of calcium carbonate. (b) Monocrystals or polycrystals of calcite present immediately below the periostracum of the L. fortunei shell. See location in the images of Figure 3. Above each image, diagrams appear (step 1 and step 2) suggesting the process of dissolution of calcium phosphate and precipitation of calcium carbonate in the calcite phase. Dissolution takes place by protonation of the carbonate group to form carbonic acid and protonation of the phosphate groups to form phosphoric acid (2).

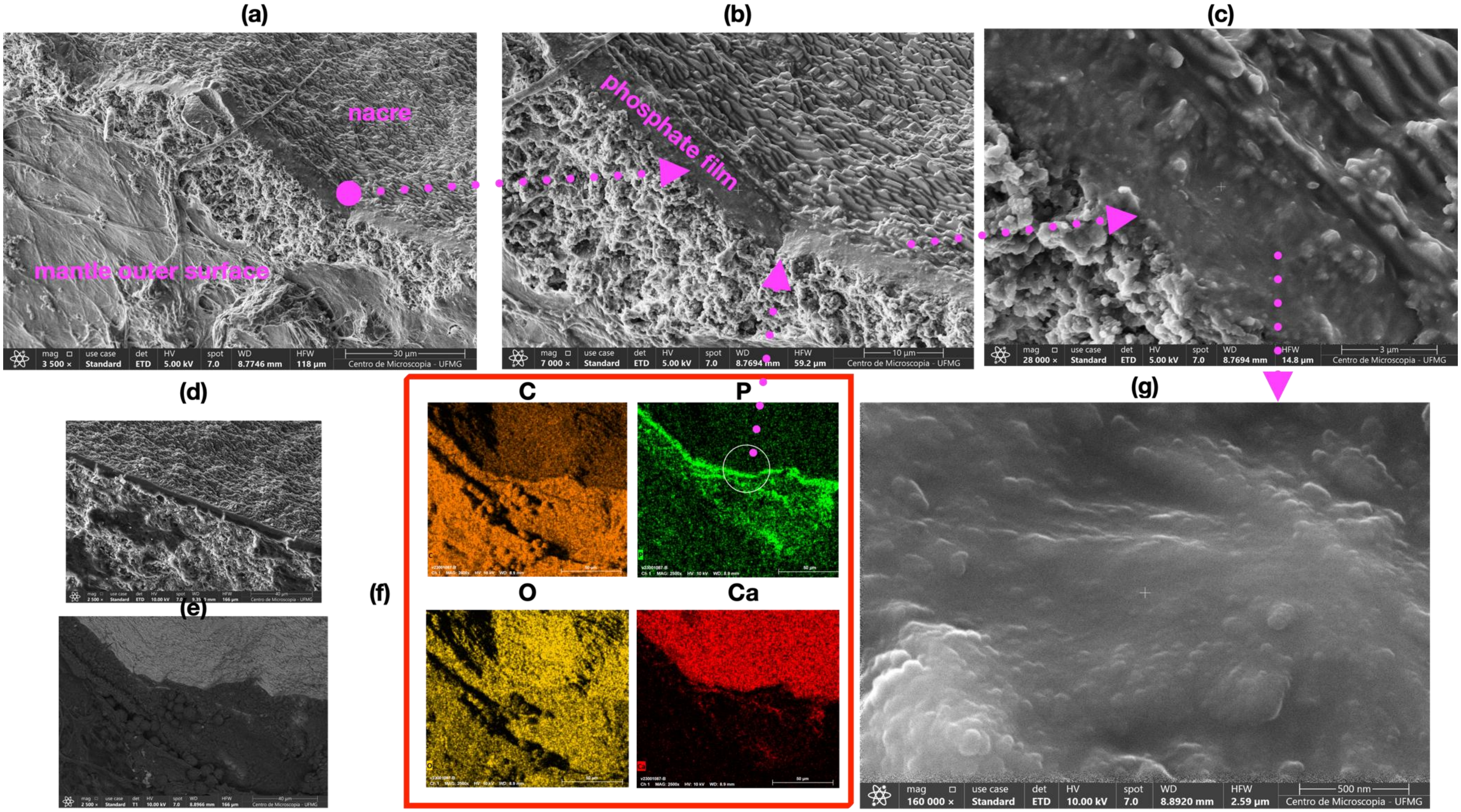

Figure S6- (a) Surface of the mantle (lower part of the image) and nacre in the upper part of the image; (b) Between the mantle and the nacre the phosphate film; (c) Image of the phosphate film in detail. (d) and (e) show the same region using different SEM sensors; (f) Maps of the four main chemical elements observed in the image: carbon (C), phosphorus (P), oxygen (O) and calcium (Ca); (g) Nanoparticles in the phosphate film.

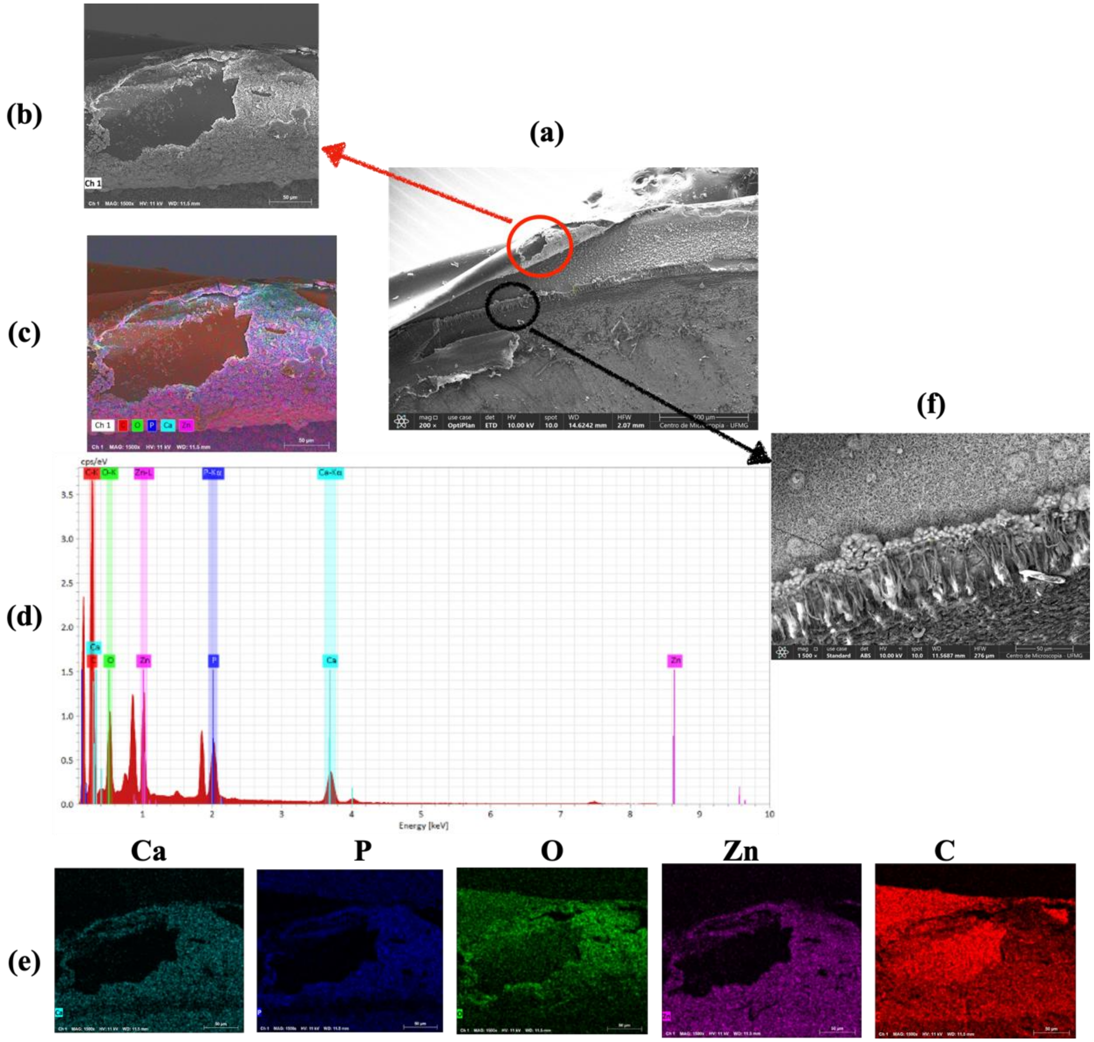

Figure S7 - (a) Sample from the edge of the L fortunei shell (SEM) was analyzed by EDS; (b) Area circled in red is shown in detail. (c) The main chemical elements found were C, Ca, P, O and Zn, as shown in the atomic concentration graph in (d). (e) The element maps show that Zn marks the region where calcium (Ca), phosphorus (P) and oxygen (O) occur. Carbon (C) does not coincide with the other elements. (f) Shows in detail the region circled in black. The morphology of the crystals in the upper part of this image is identical to that shown in figures 2, 3 and 6 of the article. The presence of zinc (Zn), a metallic element in the enzyme carbonic anhydrase (CA), was not found in other samples, only at this edge.

Carbonic Anhydrase Assay

Table S1 presents the results obtained for the carbonic anhydrase assay in demineralized suspensions of L. fortunei and the reference material bovine carbonic anhydrase (BCA).

Table S1 – Carbonic Anhydrase Assay

column / row	1	2	3	4	5	6
	**sample**	**T(s). Method 1**	**(To-T)/To (WAU unit) - method 1**	**T (s) Method 2**	**(To-T)/To (WAU unit) - method 2**	**Specific activity (WAU units/mg)**
1	Blank (To)	90	-	120	-	-
2	BCA	34	0.62	70	0.42	Method 1: 2,99 x10^3, Method 2: 2,4 x 10^3
3	DS-AA-Lf	61	0.32	70	0.42	-
4	DS-EDTA-Lf	120	-	120	-	-
5	DS-EDTA-Lf ($ZnCl_2$ added)	71	0.21	-	-	-

Method 1: reference (3); Method 2: reference (4).
T: time in seconds for the pH drop from 8.3 to 7.3 in method 1 and from pH 8.2 to 6.5 in method 2.
To: time for pH drop with buffer solution only.
Blank: buffer only.
BCA: bovine carbonic anhydrase.
DS-AA-Lf: demineralized shell solution in acetic acid.
DS-EDTA-Lf: demineralized solution with EDTA.
DS-EDTA-Lf ($ZnCl_2$ added): $ZnCl_2$ added to the demineralized solution with EDTA.
WAU: Wilbur-Anderson Units.
The results for blank, BCA, and DS-AA-Lf in method 1 are the average of six independent experiments.

The results indicate that the suspensions obtained by demineralization with acetic acid and EDTA resemble the behavior of BCA when in the presence of excess CO_2, causing a pH drop from >8 to values below 6.5. This result is due to the reaction catalyzed by carbonic anhydrase in the interconversion of CO_2 and water into a bicarbonate ion and a proton:

$$CO_2 + H_2O \leftrightarrow HCO_3^- + H^+ \quad [1]$$

Assays conducted with buffer solutions only (blank) showed much longer times, indicating the absence of the enzymatic catalyst. Assays with demineralized shell suspension with 0.5 M EDTA at pH 8.0, without the subsequent addition of $ZnCl_2$ (line 4 of Table 4), showed a time interval equal to that of the solution containing only the buffer.

Both methods involve the addition of CO_2 to the suspension. The pH in the buffer suspension also tends to acidify over time due to the increasing CO_2 concentration. However, the times are longer in the absence of carbonic anhydrase, as shown in Table 4, indicating that pH drop is slower without the presence of this enzyme.

The Wilbur-Anderson unit (WAU) value of the reference material (column 3 of Table 4), bovine carbonic anhydrase (BCA), is more than double the values obtained for the demineralized shell suspensions, indicating a higher specific enzymatic activity (column 6 of Table 4). The specific enzymatic activities of the suspensions were not calculated, as the objective of this study was to demonstrate the presence of enzymes in the shell with activity like that of carbonic anhydrase.

References

1. Constantz BR, Ison IC, Fulmer MT, Poser RD, Smith ST, VanWagoner M, et al. Skeletal repair by in situ formation of the mineral phase of bone. Science (1979). 1995;267(5205):1796–9.
2. Hankermeyer CR, Ohashi KL, Delaney DC, Ross J, Constantz BR. Dissolution rates of carbonated hydroxyapatite in hydrochloric acid. Biomaterials. 2002;23(3):743–50.
3. Miyamoto H, Miyashita T, Okushima M, Nakano S, Morita T, Matsushiro A. A carbonic anhydrase from the nacreous layer in oyster pearls. Proceedings of the National Academy of Sciences. 1996;93(18):9657–60.
4. Fuchs W, Steger F, Reich J, Ribitsch D, Rittmann SKM, Bochmann G. A simple and straightforward method for activity measurement of carbonic anhydrases. Catalysts. 2021;11(7):819.
